# Comparison of software packages for detecting unannotated translated small open reading frames by Ribo-seq

**DOI:** 10.1093/bib/bbae268

**Published:** 2024-06-06

**Authors:** Gregory Tong, Nasun Hah, Thomas F Martinez

**Affiliations:** Department of Pharmaceutical Sciences, University of California, Irvine, Irvine, CA 92617, United States; Chapman Charitable Foundations Genomic Sequencing Core, The Salk Institute for Biological Studies, La Jolla, CA 92037, United States; Department of Pharmaceutical Sciences, University of California, Irvine, Irvine, CA 92617, United States; Department of Biological Chemistry, University of California, Irvine, Irvine, CA 92617, United States; Chao Family Comprehensive Cancer Center, University of California, Irvine, Irvine, CA 92617, United States

**Keywords:** smORF annotation, Ribo-seq, microprotein, translation

## Abstract

Accurate and comprehensive annotation of microprotein-coding small open reading frames (smORFs) is critical to our understanding of normal physiology and disease. Empirical identification of translated smORFs is carried out primarily using ribosome profiling (Ribo-seq). While effective, published Ribo-seq datasets can vary drastically in quality and different analysis tools are frequently employed. Here, we examine the impact of these factors on identifying translated smORFs. We compared five commonly used software tools that assess open reading frame translation from Ribo-seq (RibORFv0.1, RibORFv1.0, RiboCode, ORFquant, and Ribo-TISH) and found surprisingly low agreement across all tools. Only ~2% of smORFs were called translated by all five tools, and ~15% by three or more tools when assessing the same high-resolution Ribo-seq dataset. For larger annotated genes, the same analysis showed ~74% agreement across all five tools. We also found that some tools are strongly biased against low-resolution Ribo-seq data, while others are more tolerant. Analyzing Ribo-seq coverage revealed that smORFs detected by more than one tool tend to have higher translation levels and higher fractions of in-frame reads, consistent with what was observed for annotated genes. Together these results support employing multiple tools to identify the most confident microprotein-coding smORFs and choosing the tools based on the quality of the dataset and the planned downstream characterization experiments of the predicted smORFs.

## Introduction

Early efforts to annotate eukaryotic genomes relied in part on applying expected properties of coding regions, such as having an AUG start codon in frame with a downstream stop codon, one protein-coding region per transcript that is often the longest open reading frame (ORF), and a minimum length cutoff of 100 codons to identify overlooked coding regions [1]. While effective, there remained the possibility that ORFs which do not follow these rules can be translated to encode functional proteins. Recent advances in genomics, proteomics, and bioinformatics have allowed researchers to empirically define protein coding regions within genomes with better precision [2–4]. The most striking result of these new studies is that thousands of small open reading frames (smORFs) containing <100–150 codons, which were presumed to be randomly occurring and non-functional, are in fact translated into small proteins dubbed microproteins [5–7]. These smORFs make up the majority of unannotated ORFs and represent an increasingly active area of research. Many microproteins have now been shown to be critical in normal biological processes and disease [8, 9].

One of the primary methods for re-annotation of genomes is based on ribosome profiling (Ribo-seq) [10–15]. Ribo-seq involves stalling elongating ribosomes in cell or tissue lysates with the small molecule inhibitor cycloheximide, followed by digestion of polysomes with an RNase and preparation of the ribosome-protected RNA fragments (RPFs) into next generation sequencing libraries [16]. Following sequencing, the resulting reads are processed and aligned to the genome to determine the locations of the ribosomes in each sample at harvesting. By identifying the locations of ribosomes, bioinformatic tools can then be applied to infer which ORFs are translated. However, due to the variation in Ribo-seq protocols and a variety of different software tools that have been developed to analyze translation from Ribo-seq data [17], there is no consensus on best practices within the field for predicting smORFs.

For the field to progress further toward functional investigation of individual microproteins and exploration of their utility as therapeutic targets, confidence in which smORFs are annotated as translated is needed. Previously, we showed that differences in Ribo-seq data quality can strongly impact which smORFs are called translated and that analyzing biological replicate datasets is helpful for separating robustly translated smORFs from noise [12]. Here, we hypothesized that different software tools for interpreting Ribo-seq data can also introduce inconsistencies into which smORFs are considered translated due to differences in the properties of Ribo-seq data that are considered in scoring, how they are weighted, and what statistical methods or classifiers are applied. To understand how the choice of software tool can influence smORF prediction, we evaluated the performances of several popular Ribo-seq-based ORF prediction tools. We found that although all tools show high congruence when identifying larger annotated ORFs as translated, they show low similarity for which unannotated smORFs are predicted to be translated. Analysis of Ribo-seq coverage levels between annotated ORFs and unannotated smORFs suggests that the overall lower translation levels of smORFs contribute to their noisier translation predictions. In addition, we observed large differences between the tools’ abilities to predict smORF translation when using lower quality Ribo-seq datasets versus high. We also demonstrated that incorporation of RNA-seq-derived de novo transcriptome assemblies can add a relatively small number of additional unannotated smORFs compared to using the GENCODE transcriptome annotation. Altogether, these results highlight the importance of using multiple tools to raise confidence in the annotation of individual ORFs for functional studies and broaden the pool of potential smORFs to test in high-throughput screens.

## Methods

### Ribo-seq data processing and alignment

Ribo-seq datasets analyzed in this study were generated in our previous study [12], and can be downloaded from the Gene Expression Omnibus database repository under accession number GSE125218. The specific Sequence Read Archive (SRA) IDs for the Ribo-seq datasets are as follows: high-resolution HeLa-S3—SRR8449578, low-resolution HeLa-S3—SRR8449575, harringtonin (TI-seq) HeLa-S3—SRR8449585, high-resolution HEK293T—SRR8449568, medium-resolution HEK293T—SRR8449567, and low-resolution HEK293T—SRR8449566.

Ribo-seq reads were processed, aligned, and filtered as described in detail in Cao *et al.* [18]. A brief description is included here as well. Trimming of 3′ adapter sequences (AGATCGGAAGAGCACACGTCT) was carried out using the FASTX-toolkit. Next, reads aligning to rRNA and tRNA sequences were filtered out using STAR with parameters –outReadsUnmapped Fastx, and the remaining reads were subsequently aligned to the GENCODE hg38 version 39 genome assembly using STAR with the following settings –outFilterMismatchNmax 2 –outFilterMultimapNmax 4 –chimScoreSeparation 10 –chimScoreMin 20 –chimSegmentMin 15 –outSAMattributes All –outSAMtype BAM SortedByCoordinate. The resulting bam file was filtered for primary alignments using samtools with the following parameters –bS –F 0X100. Next, multimappers were removed using samtools with the following parameters –bq 255. The alignment files used for RiboCode’s prepare_transcripts function require the use of the quantMode option during STAR alignment. To run RiboCode, reads were processed separately using author recommended settings to include —outfilterMismatchNmax 2 –outSAMtype BAMSortedByCoordinate –quantMode TranscriptomeSAM Genecounts –outFilterMultiMapNmax 1 –outFilterMatchNmin 16 –alignEndsType EndToEnd. Histograms of RPF length were generated by sampling a million reads and sorting by length from the final alignment file. Ribosome A-site metagene plots were created using RibORFv0.1’s readDist.pl function and a custom script was used to calculate the fraction of in-frame reads. Other tools also have the capability to generate metagene plots. To ensure the same set of read lengths were used for analysis across the different workflows, the same read lengths and offset corrections were used for all ORF predictions for each separate library. Ribo-seq coverage was visualized by generating bedgraphs using HOMER and uploading the bedgraphs to the UCSC Genome Browser.

### Tools compared in this study for microprotein-coding smORF identification

#### RibORFv0.1

RibORFv0.1 is the oldest tool of those we compared and is the tool we have used to annotate microprotein-coding smORFs in our previous studies [12, 19]. RibORFv0.1 utilizes a support vector machine classifier to select translating ORFs based on the fraction of A-site reads aligned to the correct reading frame and the read distribution uniformity over the ORF. The model uses canonical ORFs and off-frame ORFs for positive and negative controls, respectively, to train the classifier to predict smORFs. A final *P*-value score is determined based on these two properties. The authors suggest a score of $\ge$0.7 as a threshold for translation. Importantly, this tool requires the user to provide a list of ORFs to be scored and cannot use the Ribo-seq data to help identify start and stop sites. ORFs were defined using a custom java script, GTFtoFASTA [12]. Using the reference GENCODE transcriptome, all three ORFs were parsed to find the most upstream canonical ATG start codon and in frame stop. If there is no canonical start codon, then the ORF is defined from stop codon to stop codon. Running RibORFv0.1 for translation scoring, ORFs were filtered with a minimum length cutoff of 18 and minimum read coverage cutoff of 10 using the ribORF.pl script. The resulting list of ORFs was further filtered with a *P*-value cutoff of $\ge$.7, max nucleotide length cutoff of 450, and a read coverage cutoff of 10.

#### RibORFv1.0

RibORFv1.0 is an updated version of RibORF which is similar to RibORFv0.1, but importantly uses a different strategy for scoring translation. Instead of a support vector machine classifier, RibORFv1.0 uses a logistic regression model to determine the *P*-value scores. In addition, RibORFv1.0 no longer uses a prescored training set of known translated and non-translated ORFs but uses the user’s own data to train prediction parameters based on predefined positive and negative ORFs. It also parses user provided transcriptomes to identify all possible ORFs and thus does not require a user provided list. ORF scoring was processed by first running the ORFannotate.pl script with default settings. After candidate ORFs are generated, ribORF.pl was used to identify translated ORFs using default settings, including an ORF or length cutoff of 6 and a read length cutoff of 11. As with RibORFv0.1, the scored ORF list was filtered with a *P*-value cutoff of $\ge$.7 and a maximum nucleotide length cutoff of 450.

#### Ribo-TISH

Like other tools, Ribo-TISH can assess ORFs for translation using standard Ribo-seq data from samples treated with cycloheximide. In addition, it can use translation initiation sequencing (TI-seq) data from cells treated with translation initiation inhibitors, e.g. harringtonin or lactimidomycin, to identify translated ORFs either with TI-seq data alone or in combination with Ribo-seq data. For scoring, it uses a non-parametric Wilcoxon rank-sum test for its assessment of 3-nt periodicity. Ribo-TISH can also parse user provided transcriptomes to identify all possible ORFs and de novo annotate the translatome. Ribo-TISH was run with the strategy of taking the most distal start codon to stop codon with RPF coverage when defining the ORF. The predict function with the parameters –longest –altcodons TTG,CTG,GTG –seq –aaseq with a *P*-value threshold of <.05 was used. For Ribo-TISH analysis with translation initiation data, the same settings were used with the additional –t flag for the harringtonin dataset input.

#### RiboCode

RiboCode is a de novo translatome annotation software that relies solely on the 3-nt periodicity pattern. For scoring, RiboCode uses a modified Wilcoxon signed rank-sum test to assess whether the P-site density for a particular ORF is greater than the densities in the alternative reading frames. Like the other modern tools, RiboCode parses a user provided transcriptome to identify all possible ORFs for scoring. RiboCode also allows the user to input non-canonical start codons to use for defining candidate ORFs. Detection of translated ORFs was identified using the RiboCode function with the settings -l no -s ATG -A CTG,GTG,TTG -g and the default *P*-value cutoff of .05.

#### ORFquant

ORFquant is also able to de novo annotate the translatome. It uses a multitaper test to select in-frame signal showing 3-nt periodicity, similar to the older RiboTaper tool developed by the same author. This tool generates a *P*-value and a cutoff of 0.05 is used to identify translated ORFs. Importantly, ORFquant requires the average signal on each covered codon to be >50% in frame and only considers AUG start codons. ORFquant was run using the authors’ recommended settings. First, a 0.2 bit file and gtf are used to create a TxDb and Rdata file using the prepare_annotation_files function. Next, the prepare_for_ORFquant function was used to process the alignment bam file and text file containing the read lengths and cutoff for analysis. Lastly, run_ORFquant was used to take the files produced in the previous steps to score ORFs using a *P*-value threshold of .05.

### Generating lists of translated unannotated smORFs

Following scoring of translation for all ORFs by each tool, multiple filters were applied to generate a list of unannotated smORFs called translated for comparisons. First, only smORFs with a minimum length cutoff of 6 codons and maximum length cutoff of 150 codons were considered. In addition, bedtools intersect was used to remove smORFs that had over 90% overlap with CDS regions of canonical genes with the following commands, −f 0.9 -v -s. We chose to exclude smORFs that overlap fully with annotated ORFs in our analysis as they can be difficult to accurately identify by Ribo-seq, but all the tools will allow for fully internal smORFs to be scored. An additional filter using BLASTP was applied to remove potential pseudogenes and potentially missed RefSeq annotated microproteins. The settings for running the BLASTP search were -outfmt 10 -max_target_Seqs 5 -evalue 0.0001, and smORFs with BLASTP scores ≥40 were filtered out.

The smORF lists used for comparisons were standardized to include each smORF’s chromosome ID, genomic start and stop coordinates, strand information, and amino acid sequence. smORFs were considered as exactly overlapping across different tools if all these fields matched. For annotated ORFs, matching gene names were considered for overlapping across different tools. Full lists of all smORFs called translated by each tool and their overlaps are available in Supplementary Data 1.

For the analysis of additional matching smORFs when allowing for potential start site or stop site isoforms, the set of smORFs called translated by RibORFv0.1, RiboCode, ORFquant, and Ribo-TISH were each compared individually against the list of smORFs called translated by RibORFv1.0, which identified the most smORFs in both HeLa-S3 datasets. Comparing smORFs across tools can result in one-to-many or many-to-many relationships when allowing for start and stop site isoforms, where an example smORF could be considered an exact match with one particular smORF in RibORFv1.0 but also a start site or stop site match with different smORFs. Thus, these analyses were run as one-way comparisons in which we determine the number of smORFs from each tool that are considered matching to any smORF(s) in the RibORFv1.0 set when allowing for isoforms. Any smORF found to contain either a matching start site, stop site, or both (an exact match) with any number of smORFs from RibORFv1.0 was counted only once as matching. For categorization of different types of matches, any smORF with an exact matching hit in the RibORFv1.0 list with the same start site, stop site, and amino acid sequence was considered an ‘Exact Match’, and those without exact matches were categorized as either ‘Matching Stop Site’ or ‘Matching Start Site’, with prioritization given to ‘Matching Stop Site’ in cases where a smORF has both start and stop site matches in the RibORFv1.0 list.

For generating translation scores for annotated genes, RibORFv0.1 was run using a separate refFlat containing GENCODE CDS regions. For RiboCode, Ribo-TISH, ORFquant, and RibORFv1.0, annotated genes that were detected were separated out from the final list of ORFs predicted.

### Defining overlapping annotated gene and smORFs across tools

Overlaps of exactly matching annotated genes and smORFs called translated by each tool were determined using the Multiple List Comparator tool from molbiotools.com. This tool was also used to generate two-way Venn diagrams included in Supplementary Figs 7 and 8. UpSet plots showing the overlap across all five tools were generated using the R package UpSetR.

### Ribo-seq read coverage, fraction in-frame, and PhyloCSF analysis

The Ribo-seq read coverage for predicted smORFs identified by each tool was quantified alongside the top expressed isoforms for annotated genes. Coverage was quantified using HOMER’s analyzeRepeats function and normalized by transcripts per million. The fraction of reads in-frame for all ORFs was taken from RibORFv0.1 output files, which report the percent of A-site reads in all three reading frames. Average PhyloCSF scores for the 58-mammal alignment used with genome build hg38 were extracted for all smORFs from the UCSC genome browser’s PhyloCSF Track Hub.

### Nanopore long-read library preparation and sequencing

Total RNA was isolated from HeLa-S3 using the QIAGEN RNeasy kit. RNA integrity was assessed using TapeStation 4200 (Agilent), and RNA samples with RIN > 8 were used for library preparation for long read sequencing. Isolated total RNA was used to generate sequencing library following Oxford Nanopore Technologies protocol for cDNA-PCR sequencing kit. About 50 ng of total RNA was first reverse transcribed for complementary strand synthesis using strand switching primers. cDNA was PCR amplified using primers that contain 5′ tags, which enables attachment of rapid sequencing adapters. The cDNA library was loaded onto R9.4.1 flow cells according to Oxford Nanopore Technologies protocol and sequenced for 48 h with High accuracy setting on GridION system in the Salk NGS core.

### De novo transciptome assembly

For the long-read RNA-seq datasets generated using the Nanopore sequencing platform, reads were processed using the FLAIR pipeline. Reads were aligned using FLAIR align module with minimap2 and converted to a SAM file in BED12 format. FLAIR correct was used to correct misaligned splice sites using the GENCODE version 39 annotation. Finally, FLAIR collapse takes the high confidence isoforms from the corrected reads to output a gtf. Using the StringTie merge option, the FLAIR gtf was merged with the GENCODE reference gtf to create a combined non-redundant set of transcripts used for downstream analysis.

For the paired-end RNA-seq datasets generated using the Illumina sequencing platform, originally generated in Martinez *et al.* [12], fastq files were downloaded from the SRA with accession codes found in Table S1 of Supplementary Data 1 and trimmed of adapter sequences using TrimGalore. Reads were aligned using STAR with the options –runMode alignReads –sjdbOverhang 100 –runRNGseed 133 –twopassMode Basic –outSAMstrandField intronMotif –outfilterINtronMotifs Remove Noncanonical –outSAMattributes All. The resulting bam file was then sorted using samtools. For each library, StringTie was used to assemble transcripts from the sorted bam files using the guided assembly option. The assembled transcripts were then merged using the StringTie merge option with the GENCODE reference transcriptome annotation. The resulting gtf file was used as the transcriptome for downstream smORF analysis. GFFCompare was used to compare and evaluate the two transcriptome assemblies.

## Results and discussion

### Tools for detecting translated open reading frames from Ribo-seq

**Figure 1:**
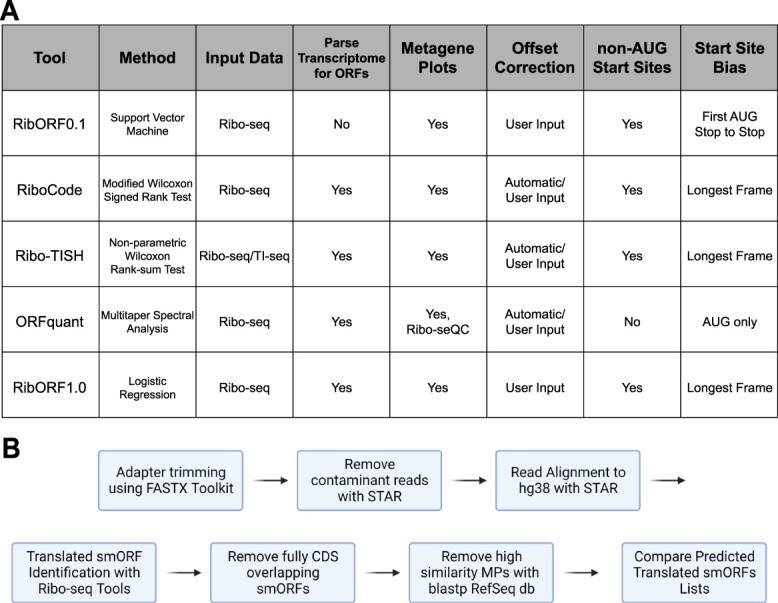
Workflow of smORF Annotation and Ribo-seq Tool Features; (A) properties of the computational methods compared in this study; (B) workflow for processing and filtering of Ribo-seq datasets that were used for ORF identification and comparison of unannotated smORFs called translated by each tool.

We compared five popular tools for analyzing individual ORFs for translation using Ribo-seq data, including RibORF version 0.1 (RibORFv0.1) [10], RibORF version 1.0 (RibORFv1.0) [20], Ribo-TISH [21], RiboCode [22], and ORFquant [23]. These tools were published between 2015 and 2020 and have been applied frequently to identify novel translated ORFs, including smORFs, in the years since. In addition, these particular tools were chosen, because each tool includes an assessment of the 3 nucleotide (3-nt) periodicity of aligned ribosomal A-site or P-site reads that are in-frame with a particular ORF to aid in scoring translation. This feature is a hallmark of active translation as the ribosome scans ORFs translating 3-nt codons from the start codon to the stop codon [16]. Higher resolution datasets have a higher percentage of reads in-frame with annotated ORFs. However, the statistical methods applied for assessing whether the fraction of in-frame reads is significant differ widely. For example, RibORFv0.1 utilizes a support vector machine approach to classify and score ORFs, while RiboCode uses a modified Wilcoxon signed-rank sum test to determine the significance in-frame versus out-of-frame read enrichment within the tested ORF (Fig. 1A). In addition, whether tools allow for ORFs initiating from near-cognate start codons, such as CUG, GUG, or UUG, or consider other features such as percent ORF coverage, differs among the tools. More details on how each tool scores translation are included in the Methods section.

To compare the tools, we developed a standardized workflow to take unprocessed Ribo-seq data and generated a filtered list of predicted novel smORFs (Fig. 1B). To summarize, 3′-adapter sequences are trimmed and reads aligning to rRNA and tRNA sequences are filtered out. The remaining reads are mapped to the hg38 genome using STAR [24] and the resulting alignment file of only uniquely mapped reads is used as the input for each tool to score ORFs for translation. Each tool was also given either a list of all possible ORFs to score, which we generated from the GENCODE comprehensive set of human transcripts, or the entire GENCODE transcriptome file for the software to parse into ORFs for scoring. The Ribo-seq datasets analyzed in our tool comparison were generated in our previous study [12] and include low- and high-resolution datasets collected from HeLa-S3 and HEK293T cell lines (Supplementary Fig. 1). The high-resolution datasets show greater than 70% in-frame RPF read alignment with known coding regions across all read lengths retained for analysis, while low-resolution data show only ~50% of in-frame RPF reads (Supplementary Fig. 2). These datasets allowed us to assess any differences between the tools in handling varying quality data and ensure that any observed trends are not cell line specific. Following scoring by each tool, smORFs that were found to fully overlap within annotated CDS regions were removed. These internal smORFs can be difficult to accurately score by Ribo-seq as reads aligned to each ORF inherently lower the score of the other ORF. The list of remaining unannotated smORFs was then used for comparison across tools.

### Comparing predicted translated smORFs across tools

In our previous study, we showed that there was a high overlap in the detection of annotated coding regions from Ribo-seq data across different resolutions, but that the set of smORFs called translated was noisy and showed low overlap across datasets [12]. This study only used RibORFv0.1 to analyze smORF translation, leaving an open question as to whether the poor overlap was an artifact of the software tool or a result of smORF translation being generally noisier and more difficult to assess relative to larger annotated coding regions.

**Figure 2:**
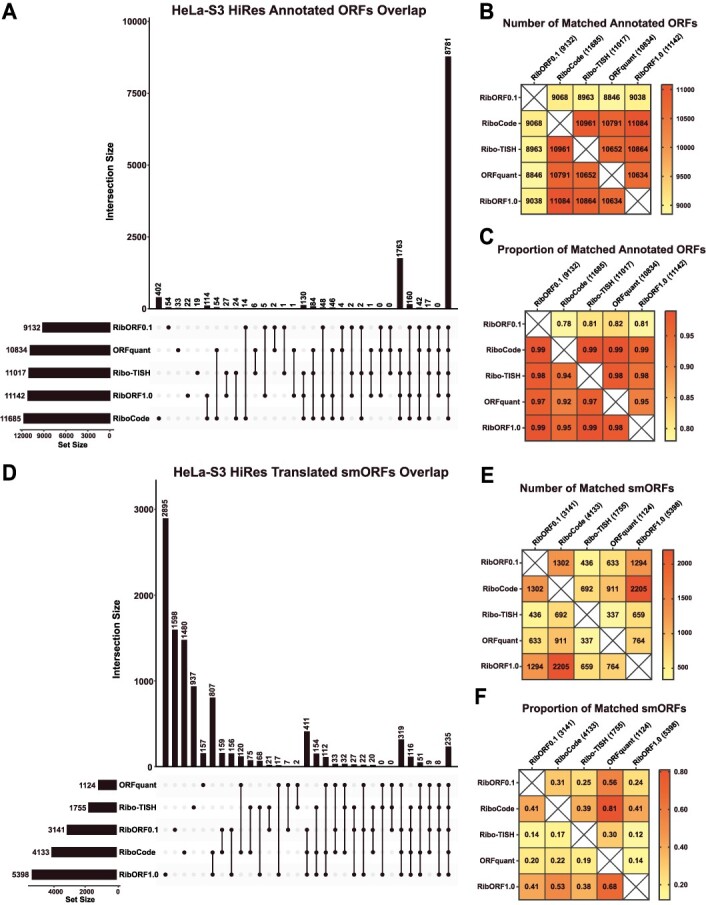
Comparison of detected annotated ORFs and predicted smORFs in the high-resolution HeLa-S3 Ribo-seq dataset; (A) UpSet plots showing the overlap of annotated genes called translated across the different tools; the total number of annotated genes detected is displayed in the bottom left bar graphs next to the names of each tool; (B) heat map showing the pairwise comparison of matching annotated genes between the different tools; (C) heat map showing the proportion of annotated genes identified by the tool in the column that are also detected by the tool in the row; (D–F) the same plots are shown as in (A–C) for the analysis of unannotated smORFs.

To answer this question, we initially examined the high-resolution HeLa-S3 Ribo-seq data for differences in identifying translated ORFs across the different tools. We observed high overlap in the number of total annotated genes detected across all five tools with 8781 (74.1%) called translated and a similar number identified by each tool (Fig. 2A). Pairwise comparisons of the number of annotated ORFs found in one tool compared to each other tool, as well as the proportion of matched ORFs, showed similar performance between all tools and that RibORFv0.1 was the least sensitive (Fig. 2B and C). Next, we examined the prediction of novel translated smORFs from each tool (Fig. 2D, Supplementary Data 1). Compared to annotated ORFs, there is little overlap in the total number of smORFs predicted with only 235 (2.3%) found across all tools and 1549 (15.4%) smORFs found in at least three out of five tools. The performance of the tools differentiated into two groups. RiboCode, RibORFv0.1, and RibORFv1.0 called 2.3–4.8 times as many smORFs translated as ORFquant and Ribo-TISH. Pairwise analysis of the number and proportion of matched smORFs revealed additional differences between the tools (Fig. 2E and F). First, despite identifying less than half the number of translated smORFs as RiboCode and RibORFv1.0, only ~40% of Ribo-TISH hits overlapped with RiboCode and RibORFv1.0. This contrasted with ORFquant, which also identified a lower amount of translated smORFs (1124) but had 68% and 81% of its calls overlap with those of RibORFv1.0 and RiboCode, respectively. In addition, Ribo-TISH had the smallest proportion of ORFquant calls matched (30%). These data demonstrate that Ribo-TISH is an outlier compared to the other tools that identify both a smaller number and a more unique set of smORFs as translated. Meanwhile, the majority of ORFquant’s hits can be captured by using the tools that predict larger numbers of translated smORFs.

**Figure 3:**
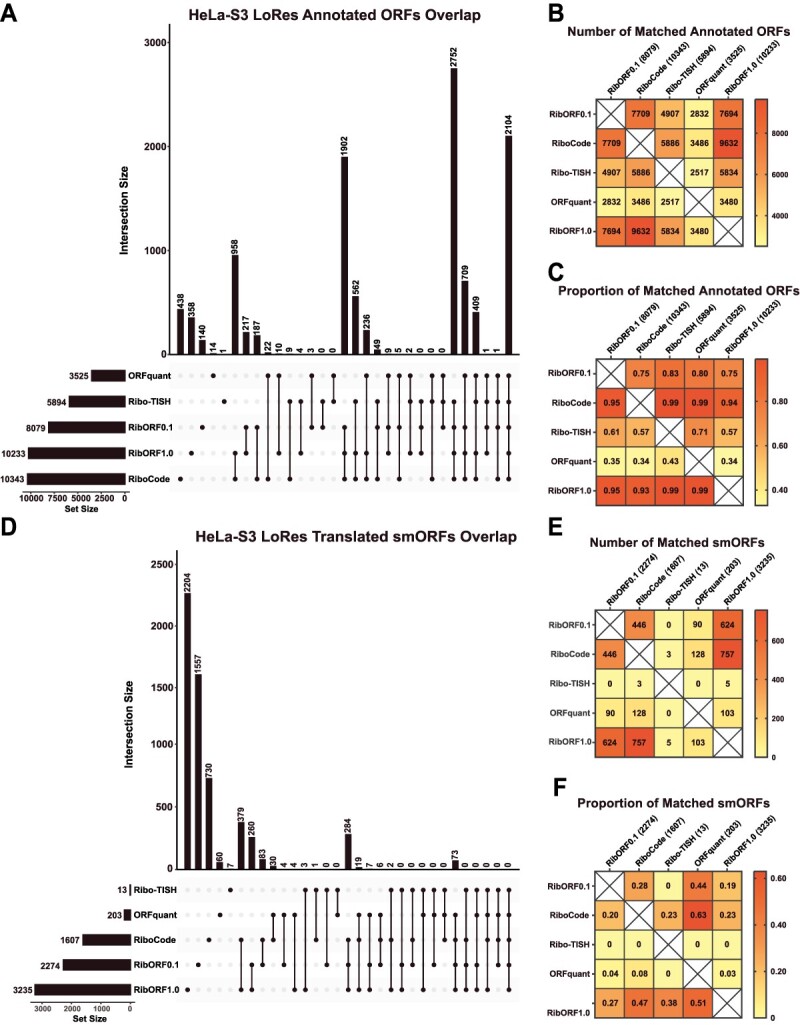
Comparison of detected annotated ORFs and predicted smORFs in low-resolution HeLa-S3 Ribo-seq dataset; (A) UpSet plots showing the overlap of annotated genes called translated across the different tools; the total number of annotated genes detected is displayed in the bottom left bar graphs next to the names of each tool; (B) heat map showing the pairwise comparison of matching annotated genes between the different tools; (C) heat map showing the proportion of annotated genes identified by the tool in the column that are also detected by the tool in the row; (D–F) the same plots are shown as in (A–C) for the analysis of unannotated smORFs.

We next explored whether these trends would remain consistent after analyzing low-resolution HeLa-S3 Ribo-seq data. Compared to the analyses using the high-resolution dataset, we observed a large drop in the number of annotated genes called translated by ORFquant (3525) and Ribo-TISH (5894), resulting in only 2104 (19%) in common across all tools (Fig. 3A). Pairwise comparisons of the tools showed that both RibORFv1.0 and RiboCode identified the most annotated genes as translated and captured >90% of those identified in all the other tools (Fig. 3B and C). ORFquant was impacted the most by the low-resolution data, identifying only 3525 annotated genes as translated. This is consistent with ORFquant’s requirement to have >50% reads in-frame for each codon within an ORF to be called translated [23]. These same observations extended to smORF prediction. Ribo-TISH and ORFquant were greatly affected by the lower resolution when predicting novel smORFs, identifying only 13 and 203 smORFs as translated, respectively (Fig. 3D, Supplementary Data 1). On the other hand, both versions of RibORF and RiboCode called many more smORFs translated and had more hits in common with each other than with Ribo-TISH and ORFquant (Fig. 3E). As observed with the high-resolution dataset, ORFquant predictions, though limited, were captured relatively well by RibORF and RiboCode (Fig. 3F).

To confirm these results, we repeated the comparison analysis for HEK293T Ribo-seq datasets with varying resolutions. We observed a similarly high degree of overlap among all tools for annotated genes scored as translated (Supplementary Fig. 3), but little overlap for unannotated smORFs called translated (Supplementary Fig. 4). We also validated the conclusions that ORFquant and Ribo-TISH are less noise tolerant and identify far fewer ORFs as translated compared to the other three pipelines when using lower resolution datasets. Furthermore, as with the HeLa-S3 datasets, both versions of RibORF called more smORFs translated than RiboCode when analyzing lower resolution datasets.

We also directly compared both annotated gene and smORF predictions across the low- and high-resolution HeLa-S3 datasets. For annotated genes, only a slightly lower number of genes were called translated when using low-resolution versus high-resolution datasets for both versions of RibORF and RiboCode, consistent with our previous study [12] (Supplementary Fig. 5A). In addition, a large proportion of genes identified in the high-resolution dataset were also captured when analyzing the low-resolution dataset with the same tool (between 87% and 91%) (Supplementary Fig. 5B). For Ribo-TISH and ORFquant, however, the proportion of genes also captured by the low-resolution dataset was much lower (between 32% and 53%). This was expected given that these tools called far fewer genes translated when analyzing the low-resolution dataset. For unannotated smORFs, similar trends between the tools were observed. For both versions of RibORF and RiboCode, the proportion of smORFs called translated using the high-resolution dataset that overlap with those detected in the low-resolution dataset was between 17% and 25% (Supplementary Fig. 5C and D). Although much lower than the overlaps for annotated genes, these were still higher than what was found for ORFquant, where the proportion of smORFs called translated across the different datasets was only ~5%. Moreover, Ribo-TISH only identified three total hits in common when comparing low- versus high-resolution datasets. These results demonstrate that RibORF and RiboCode can more sensitively detect *bona fide* translated annotated genes from low-resolution datasets than Ribo-TISH and ORFquant, and support their effectiveness for identifying at least some high-confidence smORFs from lower quality Ribo-seq data. Nevertheless, higher resolution data are expected to produce more accurate translation calls for all tools.

### Accounting for isoform differences in smORF predictions

**Figure 4:**
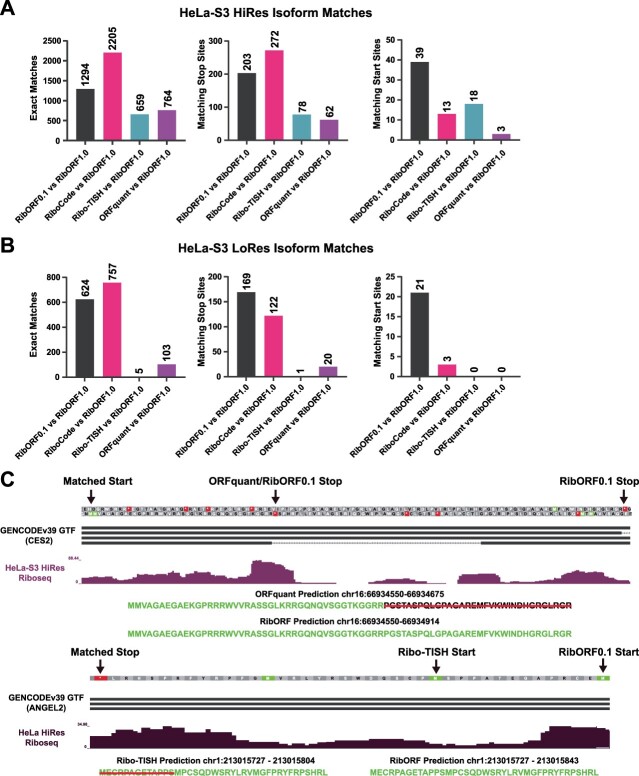
Accounting for smORF isoform variance across the different tools; (A and B) bar plots showing the number of exact smORF matches (left), additional start site isoform smORF matches (middle), and additional stop site isoform smORF matches (right) between each tool and RibORFv1.0 when analyzing either the high-resolution (A) or low-resolution (B) HeLa-S3 Ribo-seq dataset; (C) bedgraph tracks showing Ribo-seq coverage on the 5′-UTRs of CES2 and ANGEL2. In the top track, alternatively spliced smORFs on the positive strand were identified by both ORFquant and RibORFv0.1 with matching start sites but different the stop sites; in the bottom track, Ribo-TISH and RibORFv0.1 detect a smORF on the negative strand with the same stop location but different canonical start codons.

In our initial comparisons between the tools, we restricted the matches to smORFs that have the same genomic coordinates. However, given that smORFs can use alternative start codons and be spliced like larger ORFs, it is possible that the tools predict isoforms of the same smORF. To account for this, we looked for any additional smORFs identified by each tool that have the same start coordinate but different stop coordinates and vice versa using our HeLa-S3 datasets. Each tool was pairwise compared against RibORFv1.0, which predicted the largest number of smORFs. For the high-resolution dataset, allowing for stop site matches (start site isoforms) resulted in an additional 62 to 272 smORFs in common, while allowing for start site matches (stop site isoforms) resulted in an additional 3 to 39 smORFs in common (Fig. 4A, Supplementary Data 2). The high number of start site isoforms called by the different tools is expected due to how the different pipelines handle AUG versus near cognate start codons, as well as ORFs where multiple possible start codons are present. For example, we set ORFquant to only consider canonical AUG start codons consistent with how its developers used the tool [23]. Importantly, the additional smORFs considered matching when allowing for start and stop site isoforms did not account for the majority of different smORFs called translated between tools. For instance, the number of total smORFs called translated by RiboCode using the high-resolution HeLa-S3 dataset increased from 2205 when only considering exact matches to 2490 when allowing for isoforms. Thus, out of the 4133 smORFs called translated by RiboCode (Fig. 2A), 1643 (~40%) do not match any RibORFv1.0 smORF even when considering isoforms. Similar trends were observed for the other tools when compared to RibORFv1.0. For the low-resolution HeLa-S3 dataset, additional matching smORFs were found for RibORFv0.1 and RiboCode, but very few additional hits were observed for ORFquant and Ribo-TISH due to the overall lower number of smORFs called translated by these tools (Fig. 4B). Furthermore, as with the high-resolution dataset, the additional smORFs considered matching when allowing for isoforms did not account for the majority of differences between tools.

Examples of predicted smORF isoforms that have matched start or stop coordinates can be observed in the 5′-UTRs of CES2 and ANGEL2, respectively (Fig. 4C). Ribo-seq A-site plots, which display Ribo-seq reads by reading frame and show which individual codons have coverage, show that both CES2 smORF isoforms are supported by in-frame read coverage (Supplementary Fig. 6A). However, it is interesting to note that the fraction of reads in-frame for the longer spliced version is lower after the splice site, which might explain why this isoform was not called by ORFquant. For the ANGEL2 smORFs, A-site plots show that both start sites are supported by in-frame Ribo-seq reads (Supplementary Fig. 6B).

### Incorporating TI-Seq data into smORF prediction

To aid in the prediction of novel ORFs, some newer tools like Ribo-TISH allow integration of TI-seq. TI-seq is a modified version of Ribo-seq that includes a short pretreatment with translation initiation inhibitors such as harringtonin or lactimidomycin in order to enrich for ribosome coverage on ORF start sites, providing additional evidence of their translation [2]. Using matched TI-seq HeLa-S3 data from harringtonin treated cells, we compared annotated genes and smORFs called translated when using both the TI-seq and high-resolution HeLa-S3 datasets to those identified using the high-resolution dataset alone. There was a high overlap of annotated genes detected (~73%), though fewer total genes were called translated when TI-seq data were included due to the extra requirement of having an initiation peak (Supplementary Fig. 7A). For smORFs, the overlap between the two analyses was much lower (~10%, Supplementary Fig. 7B, Supplementary Data 3). In some instances, the lack of overlap was due to different translation start sites predicted based on whether TI-seq data were incorporated or not. We highlight one example of two smORF isoforms on the TXNRD1 transcript, with one smORF starting at an AUG start codon that shows enrichment by TI-seq and the other starting at an upstream near cognate start codon that is predicted when using the high-resolution HeLa-S3 Ribo-seq dataset alone (Supplementary Fig. 7C). The accompanying A-site plot shows poorer in-frame coverage for the longer non-AUG initiated isoform, supporting the shorter AUG-initiated isoform called by TI-seq. Although differing start site predictions can explain some of the differences, some of the smORFs identified by Ribo-seq alone using Ribo-TISH might in fact not be translated since they did not show start site enrichment by TI-seq. Ribo-TISH also predicts unique smORFs found only with the integration of initiation site data, such as the smORF within the 5′-UTR of the PIGW transcript (Supplementary Fig. 7D). Thus, the inclusion of initiation site data can introduce another variable to smORF predictions.

### Impact of de novo assembled transcriptome annotation on smORF identification

**Figure 5:**
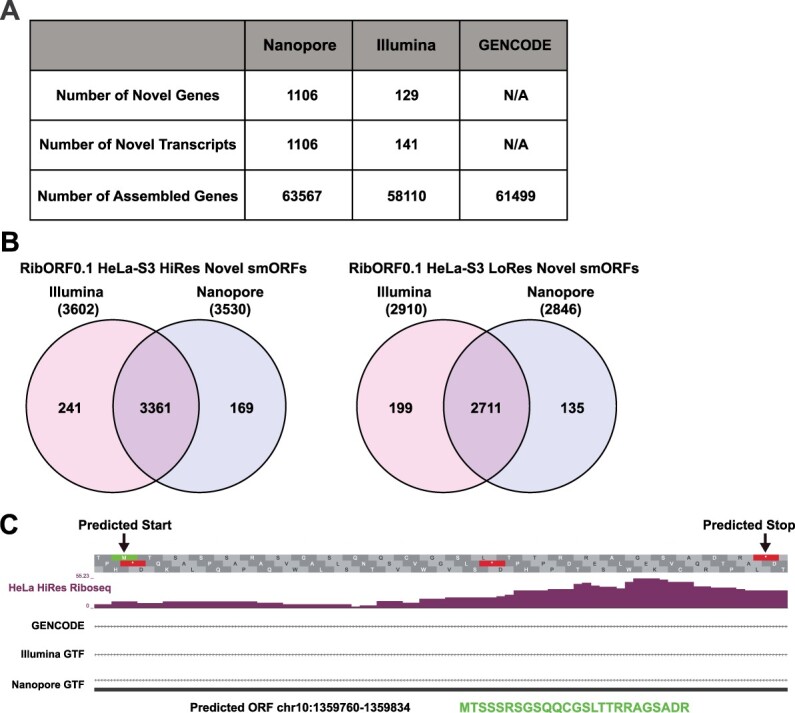
De novo transcriptome assembly enables additional smORFs to be called translated; (A) comparison of the Nanopore long read- and Illumina short read-based de novo assembled transcriptomes and GENCODE reference using GffCompare; (B) Venn diagram showing the overlap of predicted smORFs identified by RibORFv0.1 when using the de novo transcriptome assemblies along either high-resolution (left) or low-resolution (right) HeLa-S3 Ribo-seq datasets; the total number of annotated genes detected using each assembly is shown in parentheses; (C) bedgraph tracks showing Ribo-seq coverage for a smORF on the positive strand located within a region antisense to ADARB2; transcripts present in GENCODE and the de novo transcriptome assembles are also shown below. An assembled transcript for this region is only found when using the Nanopore-based de novo assembly.

Analyzing Ribo-seq data for translated smORFs requires the use of a transcriptome to create a database of all possible smORFs present in a given sample. Although most studies use transcriptomes sourced from reference databases like GENCODE [25] or Ensembl [26], de novo assembled transcriptomes can also be used. By incorporating de novo transcriptome assemblies, one can identify smORFs on transcript isoforms that are otherwise missing from these public reference databases. We previously used transcriptomes assembled from Illumina-based short read RNA-seq data to identify smORFs on cell line specific transcript isoforms [12], but use of long-read sequencing technologies may aid in the identification of additional smORFs. To evaluate the two sequencing methods’ effects on smORF identification, we assembled HeLa-S3 transcriptomes from both Nanopore long-read and Illumina short-read RNA-seq datasets using StringTie [27], a more modern assembly tool than what we had used in our original study. After assembly, the resulting transcriptome was merged with the GENCODE reference to create a comprehensive transcriptome that includes additional transcripts identified by each RNA-seq strategy. This resulted in an additional 141 transcripts using Illumina RNA-seq data and an additional 1106 transcripts using Nanopore RNA-seq data that were not included in the GENCODE transcriptome (Fig. 5A). Using RibORFv0.1 to identify translated smORFs in the high-resolution HeLa-S3 dataset with each de novo assembled transcriptome revealed a high degree of overlap (~89%, Fig. 5B, Supplementary Data 4). However, unique predicted translated smORFs were found for each transcriptome, with 241 predicted smORFs found only when using the Nanopore assembly and 169 specifically from the Illumina assembly. Using RiboCode for translation calling yielded similar results (Supplementary Fig. 8). An example smORF that both RibORFv0.1 and RiboCode called translated from a transcript specifically identified using Nanopore long-read RNA-seq data can be found antisense to ADARB2 (Fig. 5C, Supplementary Fig. 6C). These data show that incorporating de novo transcriptome assembly into smORF prediction workflows can identify additional hits, but the overall benefit over using the GENCODE reference transcriptome alone is marginal.

### Benchmarking the tools with a high confidence consensus smORF set

**Figure 6:**
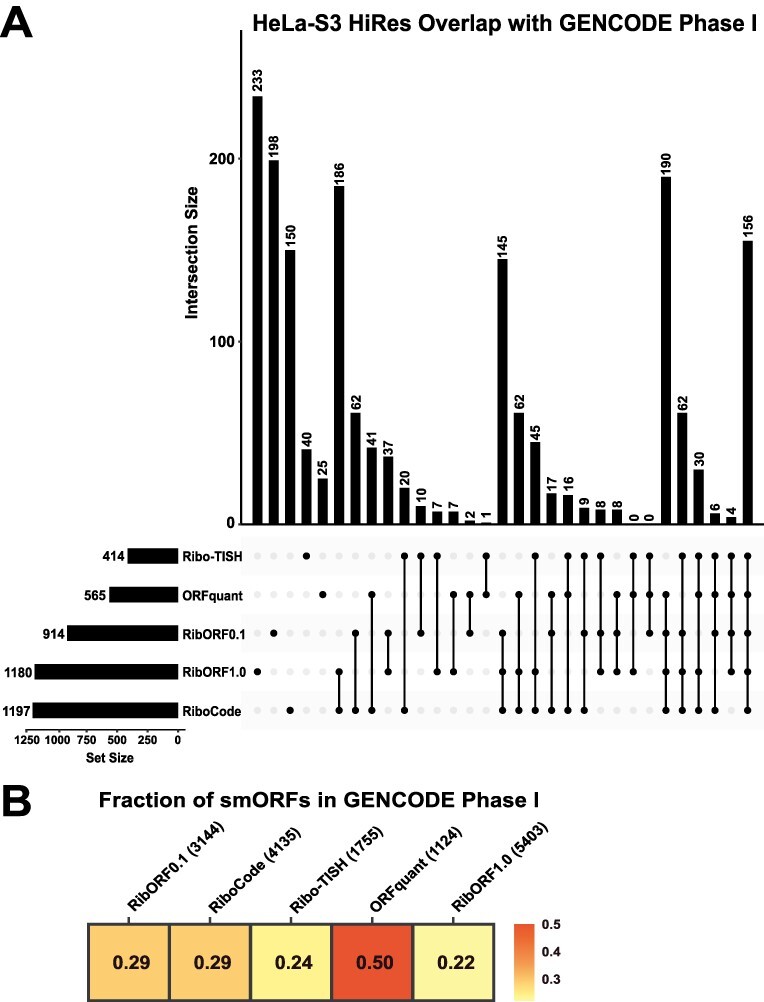
Comparison of the GENCODE Phase I high-confidence smORFs identified by each tool in the HeLa-S3 high-resolution dataset; (A) UpSet plot showing the overlap of smORFs matching the GENCODE set detected by each tool in the high-resolution HeLa-S3 Ribo-seq dataset; total number of smORFs matching the GENCODE set detected by each tool is shown in the bottom left bar graphs next to the names of each tool; for the high-resolution HeLa-S3 Ribo-seq dataset, 1797 GENCODE Phase I smORFs had 10 or more reads, representing the maximum possible number of smORFs that the tools could potentially call translated; (B) heat map showing the proportion of smORFs identified by each tool that are also included in the GENCODE smORF set.

Comparing predicted translated smORFs across tools showed high variability, leading one to question how the tools fare at identifying *bona fide* microprotein-coding smORFs. To address this point, we compared the predicted smORFs from each tool to a consensus set of 3085 smORFs that were reproducibly detected across multiple Ribo-seq-based annotation studies using different human samples and computational tools [14]. These high confidence smORF annotations are publicly available through GENCODE. Using the HeLa-S3 datasets, we determined the number of smORFs matching the GENCODE smORF set for each tool, including any potential start/stop site isoforms. For the high-resolution HeLa-S3 dataset, 156 of these high confidence GENCODE matching smORFs were predicted by all tools, and each tool was able to identify a subset of these smORFs missed by the other tools (Fig. 6A, Supplementary Data 5). RibORFv0.1, RibORFv1.0, and RiboCode had the highest number of matches, consistent with their overall greater number of smORFs called translated compared to Ribo-TISH and ORFquant. However, ORFquant had the highest proportion of its smORF calls overlap with the GENCODE set (Fig. 6B). Similar trends are observed when using the low-resolution HeLa-S3 dataset, with the exception that Ribo-TISH and ORFquant call far fewer smORFs than the other tools when using poorer quality data (Supplementary Fig. 9). Overall, these results further demonstrate that RibORF and RiboCode are more sensitive than ORFquant, while ORFquant is likely more accurate and Ribo-TISH suffers from both lower sensitivity and accuracy.

### Translation levels correlate with smORF detectability by multiple tools

Given the high overlap of annotated genes called translated across the different tools but low overlap of predicted translated smORFs, we wanted to identify properties that influence this difference. First, we found that size alone cannot explain why smORF translation prediction varies widely between tools, as each tool was able to identify a similar number of annotated ORFs <100 codons in length when analyzing the high-resolution Ribo-seq data (Supplementary Fig. 10). Next, we considered potential differences in Ribo-seq read coverage between annotated genes and smORFs, as the read coverage for a given ORF correlates with translation levels and is a critical factor in predicting translation for each of these tools. Using the high-resolution HeLa-S3 dataset, annotated genes called translated showed significantly higher read coverage than smORFs in all tools except Ribo-TISH (Supplementary Fig. 11). These same patterns were observed when analyzing the low-resolution Ribo-seq dataset using RiboCode and both RibORF versions (Supplementary Fig. 12). These data suggest that overall higher translation levels are likely driving the greater overlap in annotated gene detection across the different tools. We therefore hypothesized that smORFs that are reproducibly detected across the different tools are also more likely to have higher translation levels. Comparing smORFs called translated by all five, at least three, and less than three tools showed that smORFs detected by more tools are translated at significantly higher levels in both high- and low-resolution datasets (Supplementary Fig. 13A). These results suggest that smORFs are more difficult to detect in part because of their overall lower translation levels than larger annotated ORFs.

Another critical factor for determining translation that all tools employed in this study consider is the fraction of in-frame reads for ORFs. We therefore compared this property for smORFs called translated by all five, at least three, and less than three tools. For both the low- and high-resolution datasets, we found that smORFs called by at least three tools compared to those identified in fewer than three tools have a significantly higher fraction of in-frame reads (Supplementary Fig. 13B). These data demonstrate that smORFs called translated by more tools are not only more likely to have higher coverage, but also a greater fraction of in-frame reads, which is expected for *bona fide* translation events.

Most human microprotein-coding smORFs show conservation only to other primates or are entirely de novo occurrences in our genome [5, 14, 28, 29]. However, there are some examples of functionally characterized microproteins that are well conserved across mammals [30–34]. Therefore, we next assessed whether smORFs detected by multiple tools are not only translated at higher levels but also more conserved. Using PhyloCSF [35], a computational tool that examines evolutionary signatures that are expected for conserved coding regions, we observed no significant difference in average scores between smORFs detected by three or more tools and those detected by fewer than three tools (Supplementary Fig. 13C). Thus, conservation is not a major determinant of high confidence smORF detection by Ribo-seq. This result is consistent with only 2.4% of the GENCODE Phase I smORFs having a positive PhyloCSF score [14].

To determine whether these trends are also observed for annotated genes, we compared the coverage, fraction of in-frame reads, and PhyloCSF scores for those called translated by all five tools versus those identified by four or fewer tools (Supplementary Fig. 14). We observed significantly higher read coverage and fraction of in-frame reads, but no difference in PhyloCSF scores, for annotated genes called translated by all five tools.

## Conclusions

Ribo-seq has revolutionized our ability to de novo annotate translated ORFs. Still, it is only as effective as the bioinformatic tools used to interpret the data to accurately identify genuine translation events. By comparing several popular tools, we found that each can identify a similar set of translated annotated genes as intended when high-resolution data are used. When attempting to identify unannotated translated smORFs, however, the tools vary widely in the number called translated and show little overlap. We found a clear split between RibORFv0.1, RibORFv1.0, and RiboCode, which consistently predict more translated smORFs than ORFquant and Ribo-TISH. Moreover, RiboCode and RibORFv1.0 identify a large fraction of the same smORFs called by ORFquant, while Ribo-TISH identifies a subset of smORFs that is more unique than all the other tools. When low-resolution Ribo-seq data are used, ORFquant and Ribo-TISH are further separated from the other tools, identifying a relatively small number smORFs as translated and reflecting differences in stringency. When comparing the smORFs predicted by each tool with a high confidence set included in GENCODE, we found that RiboCode and RibORF had the highest sensitivity but ORFquant the highest accuracy. Given these results, we suggest that RiboCode and both versions of RibORF are better suited for identifying smORFs to test in high-throughput screens like CRISPR dropout assays where the aim is to identify large sets of functional smORFs. These tools are also good choices when only lower quality Ribo-seq data are available, though caution must be exercised as lower resolution data will inherently lead to noisier calls overall. ORFquant, meanwhile, is an excellent choice when attempting to identify confidently translated smORFs with AUG start sites from high-resolution data, as when planning low-throughput functional characterization studies of encoded microproteins. However, if one is interested in studying smORFs that initiate from non-AUG start sites, ORFquant is not effective. When allowing ORFquant to identify ORFs with non-AUG start sites from our high-resolution HeLa-S3 dataset, only ~0.1% of all ORFs called translated were predicted to use near cognate start sites (data not shown). Altogether, we suggest that regardless of the purpose it is prudent to use multiple Ribo-seq analysis tools in addition to analyzing biological replicates to identify the most confident microprotein-coding smORFs, particularly for ongoing annotation efforts for reference databases. Furthermore, we recommend that Ribo-seq read coverage and the fraction of reads in-frame be considered in their prioritization for downstream studies. Finally, it is our hope that these comparisons, as well as those looking at different software tools [36], will be useful to the field for developing the next generation of improved Ribo-seq interpretation tools.

**Key Points**

- Popular Ribo-seq analysis tools show little overlap in which unannotated smORFs are called translated
- Resolution of Ribo-seq data impacts both number and identity of smORFs called translated
- Higher translation levels and fraction of in-frame reads correlate with detection of smORFs by multiple tools

## Supplementary Material

Tong-et-al_Ribo-seq_Tool_Comparison_SI_FINAL-ACCEPTED_240519_bbae268

Supplementary_Data_1_bbae268

Supplementary_Data_2_bbae268

Supplementary_Data_3_bbae268

Supplementary_Data_4_bbae268

Supplementary_Data_5_bbae268

## Data Availability

Ribo-seq datasets analyzed for this study were previously published and are available from the GEO database under the accession GSE125218. Custom scripts used for processing smORF lists and settings used for all tools employed in this study are available at: https://zenodo.org/doi/10.5281/zenodo.11053751

## Author Biographies

**Gregory Tong** received his B.S. in Molecular, Cellular, and Developmental Biology from the University of Washington in 2017 and his M.S. degree in Quantitative and Systems Biology from Northwestern University in 2019. Currently, he is working toward a Ph.D. degree in Pharmaceutical Sciences at the University of California, Irvine. His current interests include bioinformatics, microproteins, and cancer-related research.

**Nasun Hah** received her B.S. and M.S. degrees in Biochemistry from Yonsei University. She then earned her Ph.D. in Biochemistry, Molecular, and Cell Biology from Cornell University in 2011. She is currently a Director of Integrative Genomics at Altos Labs.

**Thomas Martinez** received his B.S. degree in Biological Engineering from the Massachusetts Institute of Technology in 2009 and his Ph.D. in Biochemistry and Molecular Biophysics from the California Institute of Technology in 2016. He is currently an Assistant Professor of Pharmaceutical Sciences at the University of California, Irvine where his group focuses on the discovery and functional characterization of novel microproteins.

## References

[1] Basrai MA , Hieter P, Boeke JD. Small open reading frames: beautiful needles in the haystack. Genome Res 1997;7:768–71. 9267801 10.1101/gr.7.8.768

[2] Ingolia NT , Lareau LF, Weissman JS. Ribosome profiling of mouse embryonic stem cells reveals the complexity and dynamics of mammalian proteomes. Cell 2011;147:789–802. 22056041 10.1016/j.cell.2011.10.002 PMC3225288

[3] Slavoff SA , Mitchell AJ, Schwaid AG, et al. Peptidomic discovery of short open reading frame–encoded peptides in human cells. Nat Chem Biol 2013;9:59–64. 23160002 10.1038/nchembio.1120 PMC3625679

[4] Mudge JM , Jungreis I, Hunt T, et al. Discovery of high-confidence human protein-coding genes and exons by whole-genome PhyloCSF helps elucidate 118 GWAS loci. Genome Res 2019;29:2073–87. 31537640 10.1101/gr.246462.118 PMC6886504

[5] Schlesinger D , Elsässer SJ. Revisiting sORFs: overcoming challenges to identify and characterize functional microproteins. FEBS J 2022;289:53–74. 33595896 10.1111/febs.15769

[6] Wright BW , Yi Z, Weissman JS, et al. The dark proteome: translation from noncanonical open reading frames. Trends Cell Biol 2022;32:243–58. 34844857 10.1016/j.tcb.2021.10.010 PMC8934435

[7] Saghatelian A , Couso JP. Discovery and characterization of smORF-encoded bioactive polypeptides. Nat Chem Biol 2015;11:909–16. 26575237 10.1038/nchembio.1964 PMC4956473

[8] Hassel KR , Brito-Estrada O, Makarewich CA. Microproteins: overlooked regulators of physiology and disease. iScience 2023;26:106781. 37213226 10.1016/j.isci.2023.106781 PMC10199267

[9] Merino-Valverde I , Greco E, Abad M. The microproteome of cancer: from invisibility to relevance. Exp Cell Res 2020;392:111997. 32302626 10.1016/j.yexcr.2020.111997

[10] Ji Z , Song R, Regev A, et al. Many lncRNAs, 5’UTRs, and pseudogenes are translated and some are likely to express functional proteins. Elife 2015;4:e08890. 26687005 10.7554/eLife.08890 PMC4739776

[11] Van Heesch S , Witte F, Schneider-Lunitz V, et al. The translational landscape of the human heart. Cell 2019;178:242–260.e29. 31155234 10.1016/j.cell.2019.05.010

[12] Martinez TF , Chu Q, Donaldson C, et al. Accurate annotation of human protein-coding small open reading frames. Nat Chem Biol 2020;16:458–68. 31819274 10.1038/s41589-019-0425-0 PMC7085969

[13] Chen J , Brunner A-D, Cogan JZ, et al. Pervasive functional translation of noncanonical human open reading frames. Science 2020;367:1140–6. 32139545 10.1126/science.aay0262 PMC7289059

[14] Mudge JM , Ruiz-Orera J, Prensner JR, et al. Standardized annotation of translated open reading frames. Nat Biotechnol 2022;40:994–9. 35831657 10.1038/s41587-022-01369-0 PMC9757701

[15] Chothani SP , Adami E, Widjaja AA, et al. A high-resolution map of human RNA translation. Mol Cell 2022;82:2885–2899.e8. 35841888 10.1016/j.molcel.2022.06.023

[16] Ingolia NT , Ghaemmaghami S, Newman JRS, et al. Genome-wide analysis in vivo of translation with nucleotide resolution using ribosome profiling. Science 2009;324:218–23. 19213877 10.1126/science.1168978 PMC2746483

[17] Calviello L , Ohler U. Beyond read-counts: ribo-seq data analysis to understand the functions of the transcriptome. Trends Genet 2017;33:728–44. 28887026 10.1016/j.tig.2017.08.003

[18] Cao K , Hajy Heydary Y, Tong G, et al. Integrated workflow for discovery of microprotein-coding small open reading frames. STAR Protoc 2023;4:102649. 37874679 10.1016/j.xpro.2023.102649 PMC10618807

[19] Martinez TF , Lyons-Abbott S, Bookout AL, et al. Profiling mouse brown and white adipocytes to identify metabolically relevant small ORFs and functional microproteins. Cell Metab 2023;35:166–183.e11. 36599300 10.1016/j.cmet.2022.12.004 PMC9889109

[20] Ji Z . RibORF: identifying genome-wide translated open reading frames using ribosome profiling. Curr Protoc Mol Biol 2018;124:e67. 30178897 10.1002/cpmb.67 PMC6168376

[21] Zhang P , He D, Xu Y, et al. Genome-wide identification and differential analysis of translational initiation. Nat Commun 2017;8:1749. 29170441 10.1038/s41467-017-01981-8 PMC5701008

[22] Xiao Z , Huang R, Xing X, et al. De novo annotation and characterization of the translatome with ribosome profiling data. Nucleic Acids Res 2018;46:e61. 29538776 10.1093/nar/gky179 PMC6007384

[23] Calviello L , Hirsekorn A, Ohler U. Quantification of translation uncovers the functions of the alternative transcriptome. Nat Struct Mol Biol 2020;27:717–25. 32601440 10.1038/s41594-020-0450-4

[24] Dobin A , Davis CA, Schlesinger F, et al. STAR: ultrafast universal RNA-seq aligner. Bioinformatics 2013;29:15–21. 23104886 10.1093/bioinformatics/bts635 PMC3530905

[25] Frankish A , Diekhans M, Jungreis I, et al. GENCODE 2021. Nucleic Acids Res 2021;49:D916–23. 33270111 10.1093/nar/gkaa1087 PMC7778937

[26] Cunningham F , Allen JE, Allen J, et al. Ensembl 2022. Nucleic Acids Res 2022;50:D988–95. 34791404 10.1093/nar/gkab1049 PMC8728283

[27] Pertea M , Pertea GM, Antonescu CM, et al. StringTie enables improved reconstruction of a transcriptome from RNA-seq reads. Nat Biotechnol 2015;33:290–5. 25690850 10.1038/nbt.3122 PMC4643835

[28] Vakirlis N , Vance Z, Duggan KM, et al. De novo birth of functional microproteins in the human lineage. Cell Rep 2022;41:111808. 36543139 10.1016/j.celrep.2022.111808 PMC10073203

[29] Broeils LA , Ruiz-Orera J, Snel B, et al. Evolution and implications of de novo genes in humans. Nat Ecol Evol 2023;7:804–15. 36928843 10.1038/s41559-023-02014-y

[30] Rathore A , Chu Q, Tan D, et al. MIEF1 microprotein regulates mitochondrial translation. Biochemistry 2018;57:5564–75. 30215512 10.1021/acs.biochem.8b00726 PMC6443411

[31] Chu Q , Martinez TF, Novak SW, et al. Regulation of the ER stress response by a mitochondrial microprotein. Nat Commun 2019;10:4883. 31653868 10.1038/s41467-019-12816-z PMC6814811

[32] D’Lima NG , Ma J, Winkler L, et al. A human microprotein that interacts with the mRNA decapping complex. Nat Chem Biol 2017;13:174–80. 27918561 10.1038/nchembio.2249 PMC5247292

[33] Stein CS , Jadiya P, Zhang X, et al. Mitoregulin: a lncRNA-encoded microprotein that supports mitochondrial supercomplexes and respiratory efficiency. Cell Rep 2018;23:3710–3720.e8. 29949756 10.1016/j.celrep.2018.06.002 PMC6091870

[34] Zhang Q , Vashisht AA, O’Rourke J, et al. The microprotein Minion controls cell fusion and muscle formation. Nat Commun 2017;8:15664. 28569745 10.1038/ncomms15664 PMC5461507

[35] Lin MF , Jungreis I, Kellis M. PhyloCSF: a comparative genomics method to distinguish protein coding and non-coding regions. Bioinformatics 2011;27:i275–82. 21685081 10.1093/bioinformatics/btr209 PMC3117341

[36] Prensner JR , Abelin JG, Kok LW, et al. What can Ribo-Seq, immunopeptidomics, and proteomics tell us about the noncanonical proteome? Mol Cell Proteomics 2023;22:100631. 37572790 10.1016/j.mcpro.2023.100631 PMC10506109

