# Supplementary material for: Comparison of software packages for detecting unannotated translated small open reading frames by Ribo-seq
Source: Brief Bioinform. 2024 Jun 6;25(4):bbae268. doi: 10.1093/bib/bbae268 (PMC11155197; doi:10.1093/bib/bbae268)
Supplement: Tong-et-al_Ribo-seq_Tool_Comparison_SI_FINAL-ACCEPTED_240519_bbae268 [file tong-et-al_ribo-seq_tool_comparison_si_final-accepted_240519_bbae268.pdf]

SUPPLEMENTARY INFORMATION

Comparison of software packages for detecting unannotated translated small open reading frames by Ribo-seq

Gregory Tong[1], Nasun Hah[2], Thomas F. Martinez[1,3,4,*]

[1]Department of Pharmaceutical Sciences, University of California, Irvine, CA 92617, USA
[2]Chapman Charitable Foundations Genomic Sequencing Core, The Salk Institute for Biological Studies, La Jolla, CA, USA.
[3]Department of Biological Chemistry, University of California, Irvine, CA 92617, USA
[4]Chao Family Comprehensive Cancer Center, University of California, Irvine, CA 92617, USA
*Correspondence: t.martinez@uci.edu

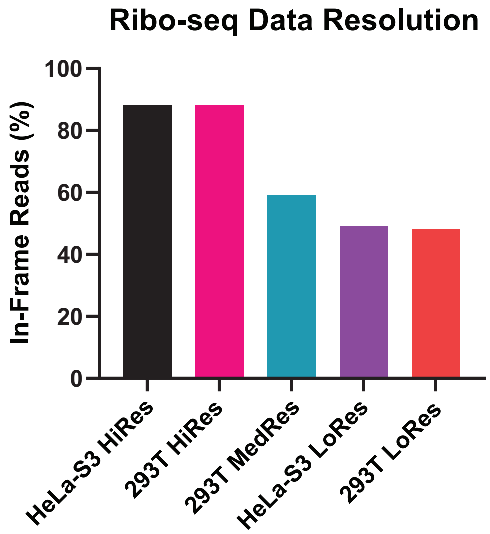

Supplementary Figure 1. Percentage of in-frame reads for the highest abundance RPF read length for each Ribo-seq dataset. For each Ribo-seq dataset analyzed, the fraction of in-frame reads after the start site was calculated for the most abundant RPF read length after offset correction to align to the ribosomal A-site. The read lengths analyzed for each dataset were: HeLa-S3 HiRes - 28 nt, 293T HiRes - 28 nt, 293T MedRes - 30 nt, HeLa-S3 LowRes – 32 nt, and 293T LowRes - 31 nt.

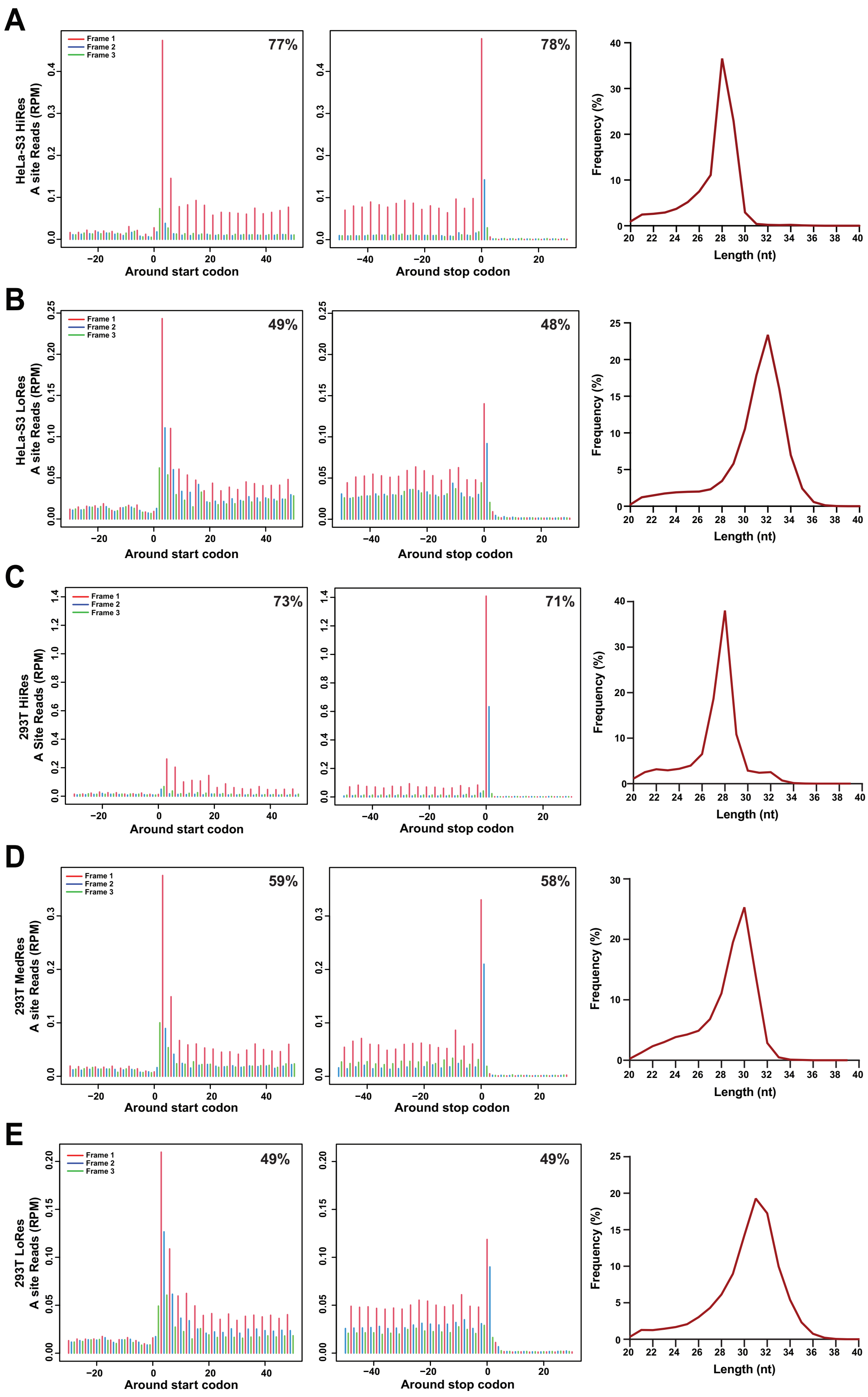
A
B
C
D
E
Frame 1
Frame 2
Frame 3
HeLa-S3 HiRes
A site Reads (RPM)
HeLa-S3 LoRes
293T HiRes
A Site Reads (RPM)
293T MedRes
293T LoRes
77%
78%
49%
48%
73%
71%
59%
58%
49%
49%
Around start codon
Around stop codon
Frequency (%)
Length (nt)

Supplementary Figure 2. Quality control of HeLa-S3 Ribo-seq datasets. (A) Metagene plots for the high-resolution HeLa-S3 dataset displaying the A-site read distribution around the start and stop sites (left). The 5'-end of each RPF read was adjusted to the ribosomal A-site after mapping to hg38 canonical genes. The coding regions are in reading frame 1, while reading frames 2 and 3 are out of frame. Line graph of the RPF read length frequency distribution peaks at 28 nt (right). Read lengths 25-29 nt were used for downstream analysis **(B)** Metagene plots for the low-resolution HeLa-S3 dataset (left). Line graph of the RPF read length frequency distribution peaks at 32 nt (right). Read lengths 31-35 nt were used for downstream analysis. **(C)** Metagene plots for the high-resolution HEK293T dataset (left). Line graph of the RPF read length frequency distribution peaks at 28 nt (right). Read lengths 25-29 nt were used for downstream analysis. **(D)** Metagene plots for the medium-resolution HEK293T dataset (left). Line graph of the RPF read length frequency distribution peaks at 30 nt (right). Read lengths 29-33 nt were used for downstream analysis. **(E)** Metagene plots for the low-resolution HEK293T dataset (left). Line graph of the RPF read length frequency distribution peaks at 31 nt (right). Read lengths 28-34 nt were used for downstream analysis.

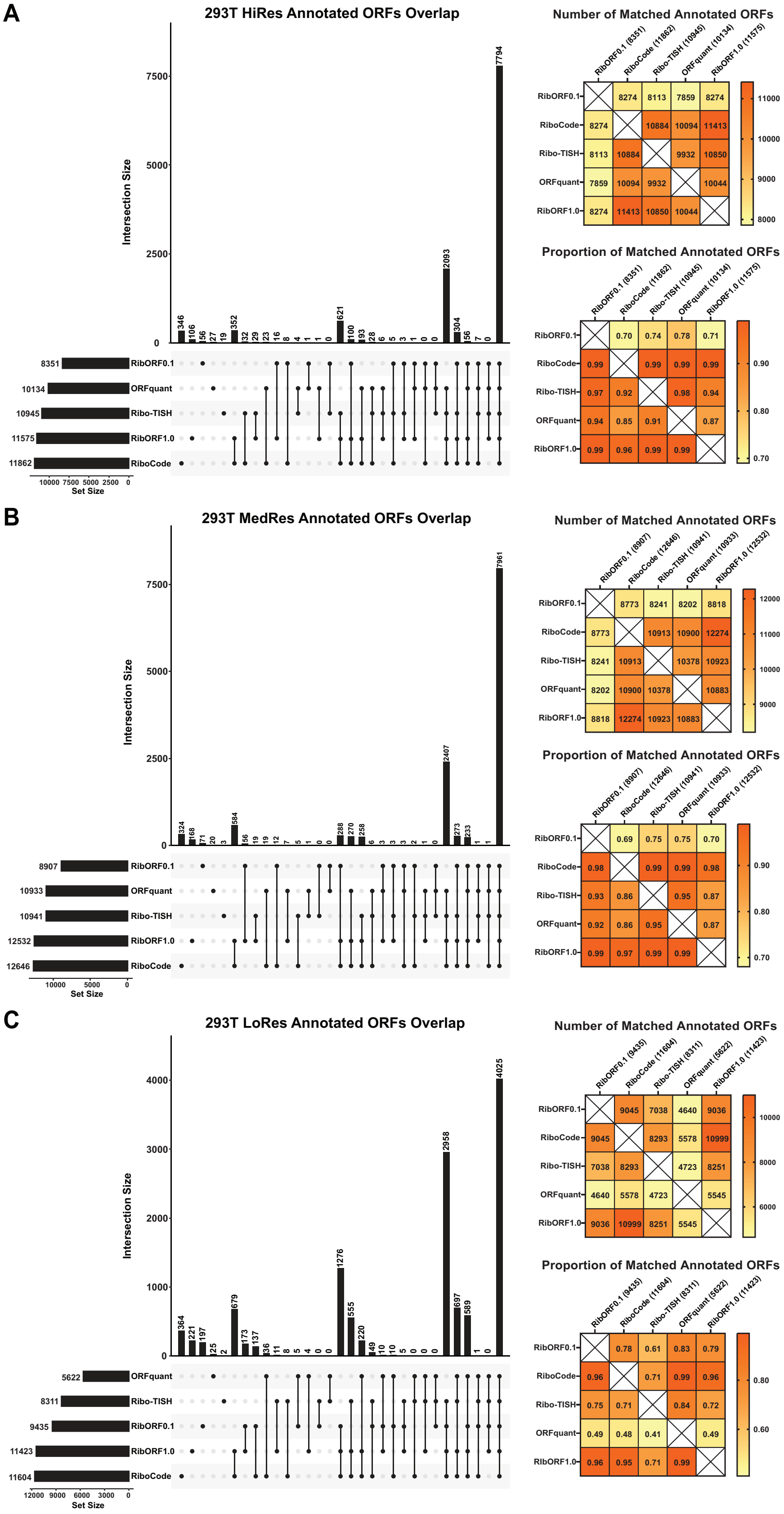
A
293T HiRes Annotated ORFs Overlap
Intersection Size
Set Size
RibORF0.1
ORFquant
Ribo-TISH
RibORF1.0
RiboCode
Number of Matched Annotated ORFs
RibORF0.1 (8351)
RiboCode (11862)
Ribo-TISH (10945)
ORFquant (10134)
RibORF1.0 (11575)
RibORF0.1 8274 8113 7859 8274
RiboCode 8274 10884 10094 11413
Ribo-TISH 8113 10884 9932 10850
ORFquant 7859 10094 9932 10044
RibORF1.0 8274 11413 10850 10044
Proportion of Matched Annotated ORFs
RibORF0.1 0.70 0.74 0.78 0.71
RiboCode 0.99 0.99 0.99 0.99
Ribo-TISH 0.97 0.92 0.98 0.94
ORFquant 0.94 0.85 0.91 0.87
RibORF1.0 0.99 0.96 0.99 0.99
B
293T MedRes Annotated ORFs Overlap
Intersection Size
Set Size
Number of Matched Annotated ORFs
RibORF0.1 (8907)
RiboCode (12646)
Ribo-TISH (10941)
ORFquant (10933)
RibORF1.0 (12532)
RibORF0.1 8773 8241 8202 8818
RiboCode 8773 10913 10900 12274
Ribo-TISH 8241 10913 10378 10923
ORFquant 8202 10900 10378 10883
RibORF1.0 8818 12274 10923 10883
Proportion of Matched Annotated ORFs
RibORF0.1 0.69 0.75 0.75 0.70
RiboCode 0.98 0.99 0.99 0.98
Ribo-TISH 0.93 0.86 0.95 0.87
ORFquant 0.92 0.86 0.95 0.87
RibORF1.0 0.99 0.97 0.99 0.99
C
293T LoRes Annotated ORFs Overlap
Intersection Size
Set Size
Number of Matched Annotated ORFs
RibORF0.1 (9435)
RiboCode (11604)
Ribo-TISH (8311)
ORFquant (5622)
RibORF1.0 (11423)
RibORF0.1 9045 7038 4640 9036
RiboCode 9045 8293 5578 10999
Ribo-TISH 7038 8293 4723 8251
ORFquant 4640 5578 4723 5545
RibORF1.0 9036 10999 8251 5545
Proportion of Matched Annotated ORFs
RibORF0.1 0.78 0.61 0.83 0.79
RiboCode 0.96 0.71 0.99 0.96
Ribo-TISH 0.75 0.71 0.84 0.72
ORFquant 0.49 0.48 0.41 0.49
RibORF1.0 0.96 0.95 0.71 0.99

Supplementary Figure 3. Comparison of detected annotated genes in HEK293T Ribo-seq datasets of varying resolution. (A-C) UpSet plots showing the overlap of annotated genes called translated across the different tools (left). The total number of annotated genes detected is displayed in the bottom left bar graphs next to the names of each tool. Heat map showing the pairwise comparison of matching annotated genes between the different tools (right, top). Heat map showing the proportion of annotated genes identified by the tool in the column that are also detected by the tool in the row (right, bottom). HEK293T datasets analyzed are categorized by their resolution: high **(A)**, medium **(B)**, and low **(C)**.

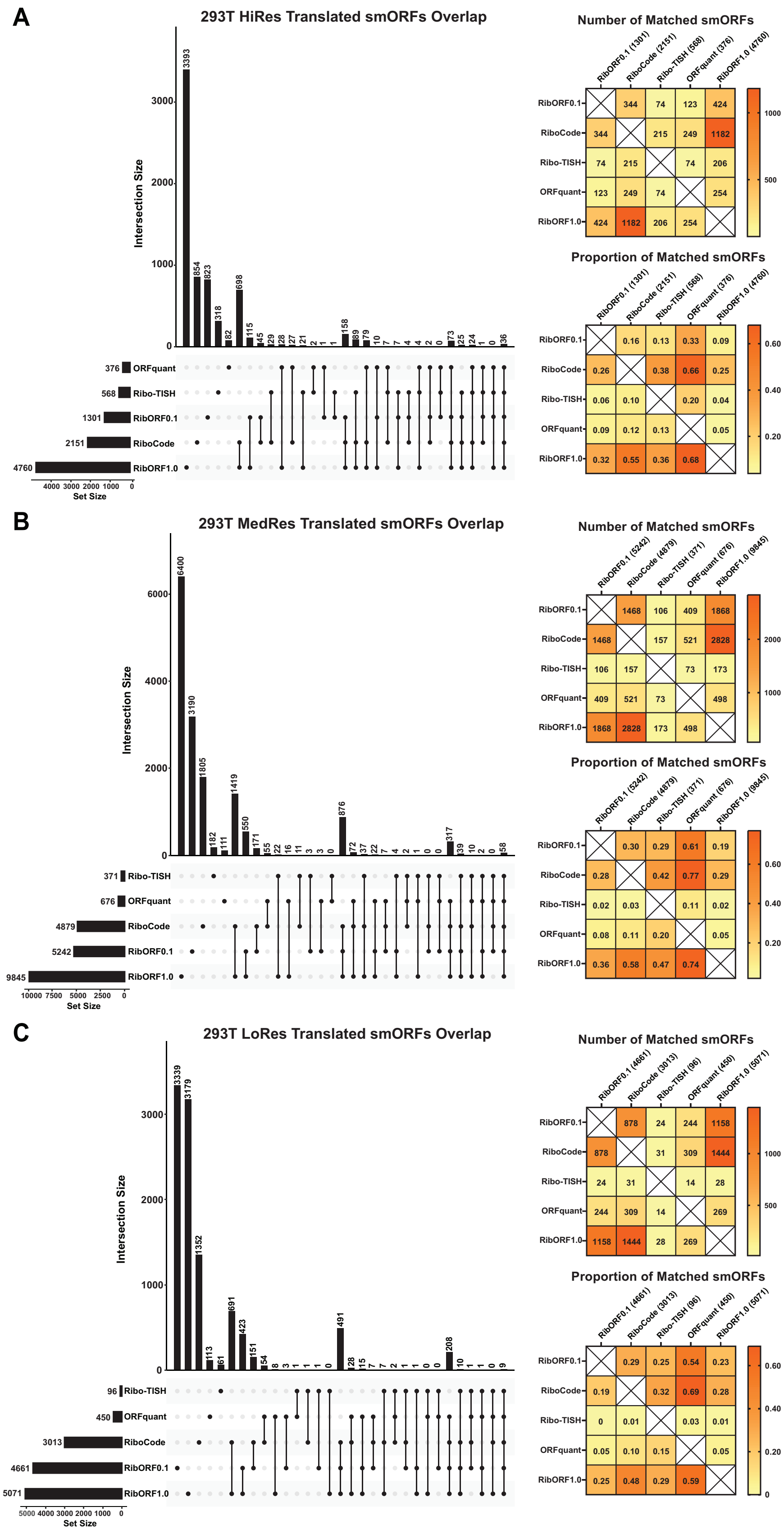

A
293T HiRes Translated smORFs Overlap
Intersection Size
Set Size
ORFquant
Ribo-TISH
RibORF0.1
RiboCode
RibORF1.0
Number of Matched smORFs
Proportion of Matched smORFs
RibORF0.1 (1301)
RiboCode (2151)
Ribo-TISH (568)
ORFquant (376)
RibORF1.0 (4760)
B
293T MedRes Translated smORFs Overlap
Intersection Size
Set Size
Number of Matched smORFs
Proportion of Matched smORFs
RibORF0.1 (5242)
RiboCode (4879)
Ribo-TISH (371)
ORFquant (676)
RibORF1.0 (9845)
C
293T LoRes Translated smORFs Overlap
Intersection Size
Set Size
Number of Matched smORFs
Proportion of Matched smORFs
RibORF0.1 (4661)
RiboCode (3013)
Ribo-TISH (96)
ORFquant (450)
RibORF1.0 (5071)

Supplementary Figure 4. Comparison of predicted smORFs in HEK293T Ribo-seq datasets of varying resolution. (A-C) UpSet plots showing the overlap of unannotated smORFs called translated across the different tools (left). The total number of smORFs detected is displayed in the bottom left bar graphs next to the names of each tool. Heat map showing the pairwise comparison of matching unannotated smORFs between the different tools (right, top). Heat map showing the proportion of unannotated smORFs identified by the tool in the column that are also detected by the tool in the row (right, bottom). HEK293T datasets analyzed are categorized by their resolution: high **(A)**, medium **(B)**, and low **(C)**.

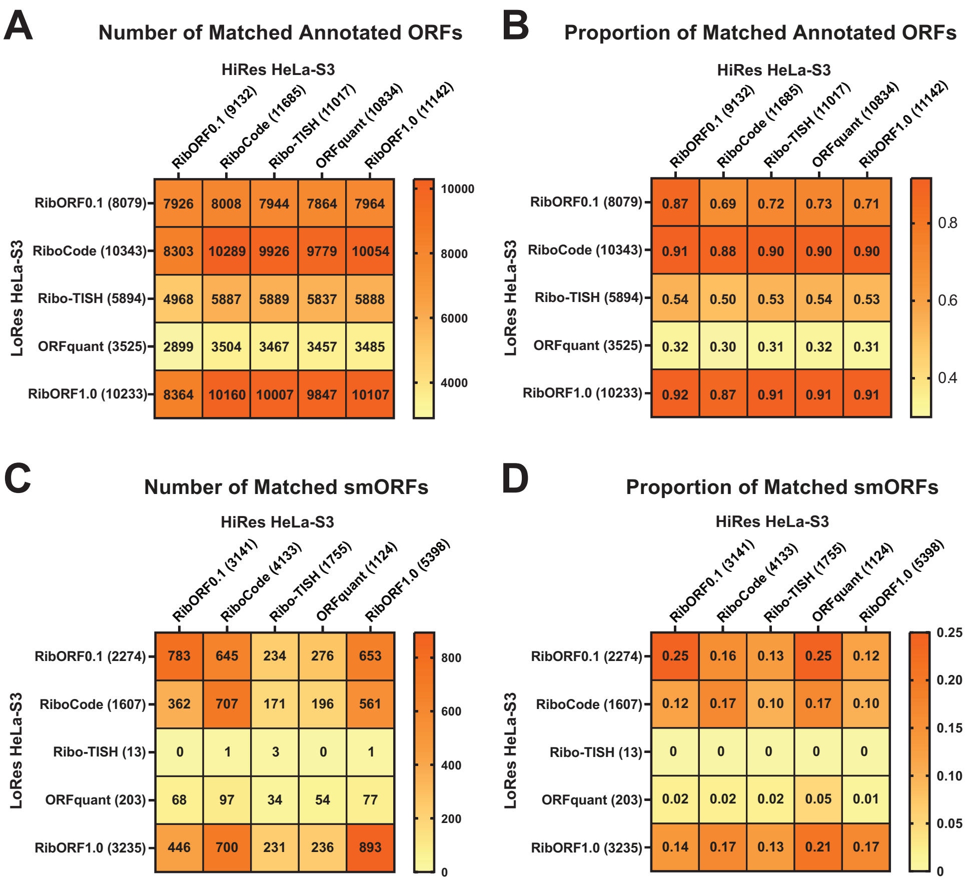

Supplementary Figure 5. Comparison of annotated genes and smORFs called translated across high- and low-resolution HeLa-S3 Ribo-seq datasets. (A) Heat map showing the pairwise comparison of matching annotated genes called translated by each tool when using low-resolution (rows) versus high-resolution (columns) HeLa-S3 Ribo-seq datasets. **(B)** Heat map showing the proportion of annotated genes identified by the tool in the column using high-resolution data that are also detected by the tool in the row using low-resolution data. The total number of smORFs detected by each tool is shown in parentheses. **(C-D)** The same plots are shown as in **(A-B)** for the analysis of unannotated smORFs.

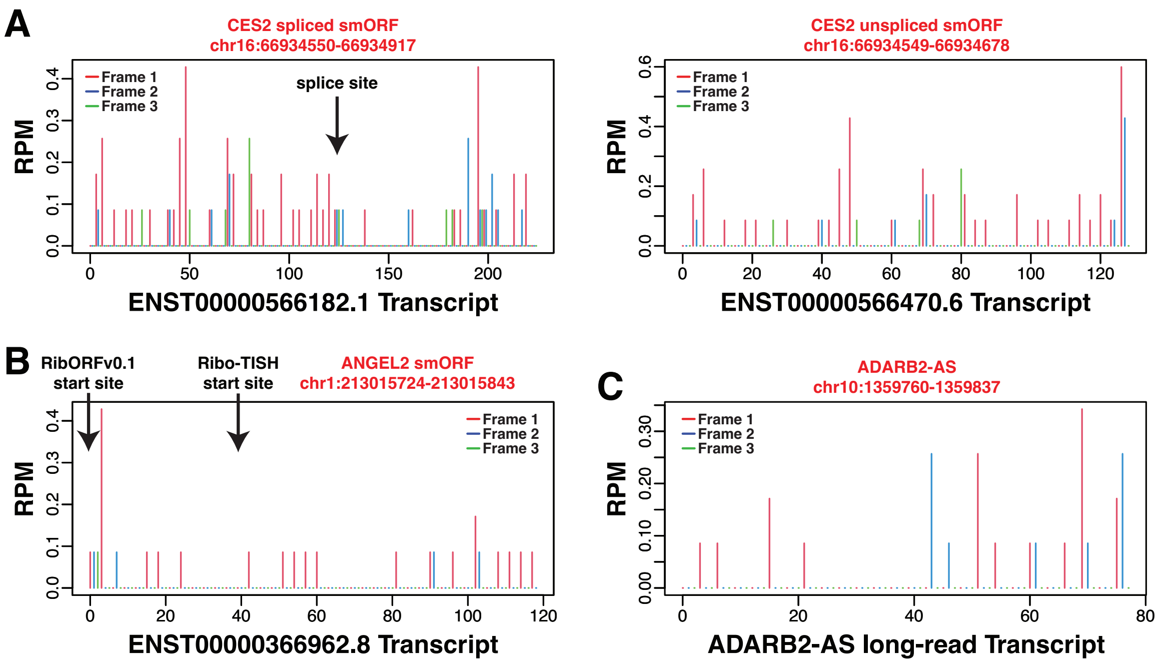

Supplementary Figure 6. Ribo-seq A-site plots for smORFs identified by different tools. **(A)** Plot of ribosome A-site reads (Ribo-Seq) for CES2 smORFs. The spliced smORF identified by RibORFv0.1 is shown on the left and the unspliced isoform identified by both RibORFv0.1 and ORFquant is shown on the right. Reads aligned to frames 1, 2, and 3 are colored red, blue, and green, respectively. RPM, reads per million. **(B)** Plot of ribosome A-site reads for ANGEL2 smORFs. Different start sites predicted by RibORFv0.1 and Ribo-TISH are shown. **(C)** Plot of ribosome A-site reads for smORF on ADARB2 anti-sense transcript assembled only when using long read RNA-seq. For all A-site plots, the smORF being considered is aligned to frame 1 (red).

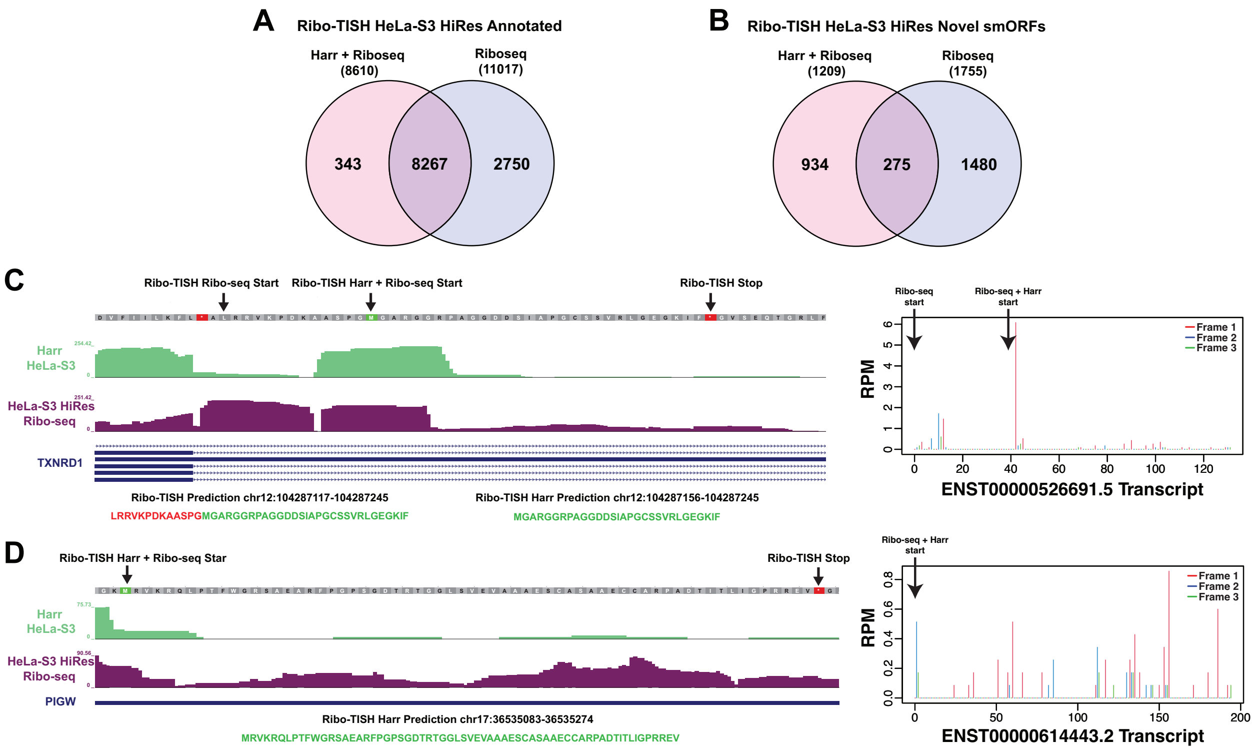

Supplementary Figure 7. Comparison of predicted smORFs using Ribo-TISH with translation initiation (TI-seq) data included and Ribo-seq data alone. (A) Venn diagram showing the overlap of annotated genes detected by Ribo-TISH including or excluding TI-seq HeLa-S3 data along with high-resolution Ribo-seq data. Total number of annotated genes identified is displayed next to each condition analyzed in parentheses. **(B)** Same analysis as in **(A)** for predicted smORFs. **(C)** Bedgraph tracks showing TI-seq and Ribo-seq coverage for smORFs called translated within the 5'-UTR of the TXNRD1 transcript on the positive strand. Both smORFs share the same stop site but have different starts called if TI-seq data is considered. Plot of ribosome A-site reads for these smORFs with different start sites highlighted is shown on the right. (**D**) Bedgraph tracks showing a smORF identified within the 5'-UTR of PIGW only when running Ribo-TISH with both TI-seq and Ribo-seq data. Plot of ribosome A-site reads for this smORF is shown on the right, and the smORF being considered is aligned to frame 1 (red).

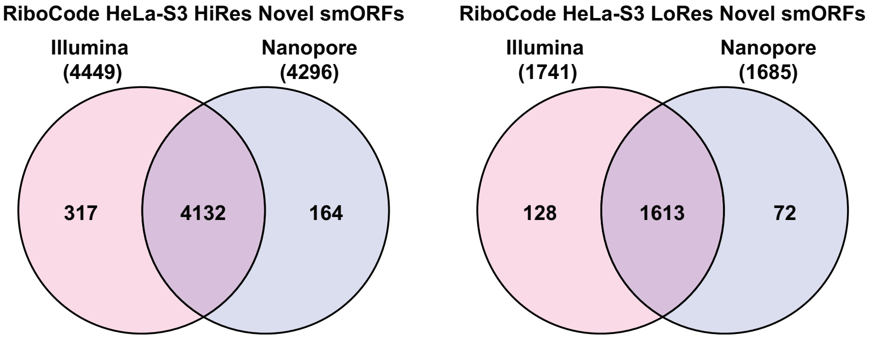

Suplementary Figure 8. Comparison of smORFs predicted by RiboCode when incorporating Nanopore- and Illumina-based *de novo* assembled transcriptomes. Venn diagram showing the overlap of predicted smORFs identified by RiboCode when using the *de novo* transcriptome assemblies along with either high-resolution (left) or low-resolution (right) HeLa-S3 Ribo-seq datasets.

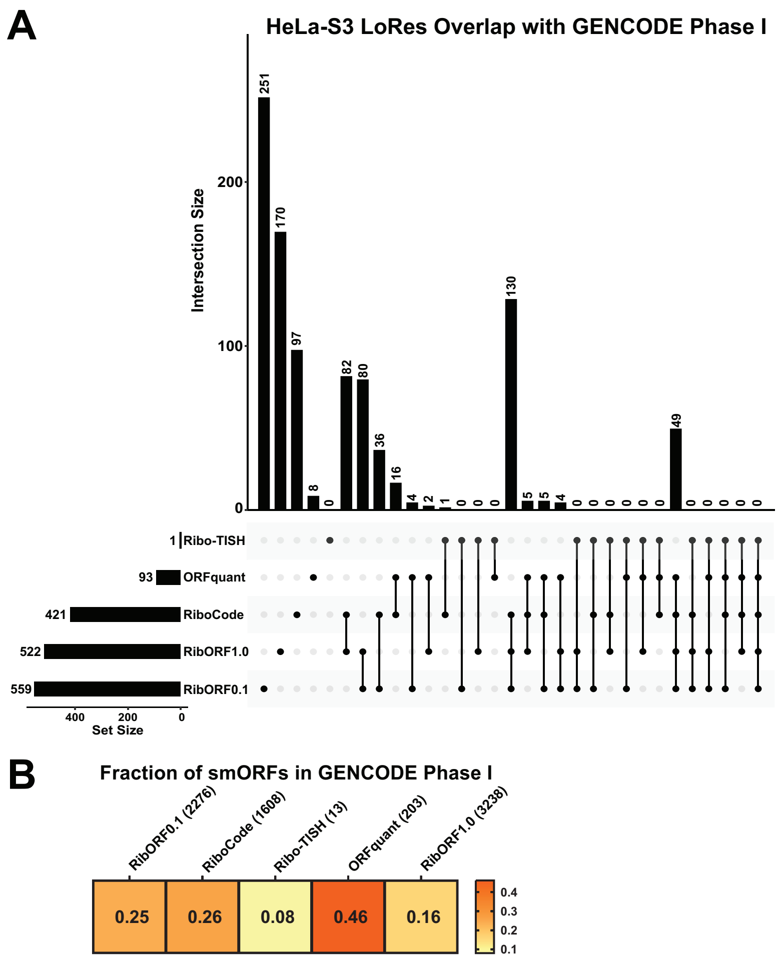

Supplementary Figure 9. Comparison of the GENCODE Phase I high-confidence smORFs predicted by each tool in the HeLa-S3 low-resolution dataset. (A) UpSet plot showing the overlap of smORFs matching the GENCODE set detected by each tool in the low-resolution HeLa-S3 Ribo-seq dataset. Total number of smORFs matching the GENCODE set detected by each tool is shown in the bottom left bar graphs next to the names of each tool. For the low-resolution HeLa-S3 Ribo-seq dataset, 1,780 GENCODE Phase I smORFs had 10 or more reads, representing the maximum possible number of smORFs that the tools could potentially call translated. **(B)** Heat map showing the proportion of smORFs identified by each tool that are also included in the GENCODE smORF set.

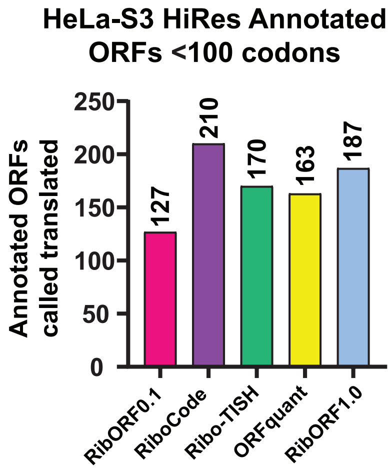

Supplementary Figure 10. Number of annotated ORFs less than 100 codons in length called translated by each tool. Bar graphs showing the number of annotated ORFs less than 100 codons in length that are detected by each tool (out of 804 total ORFs) for the high-resolution HeLa-S3 Ribo-seq dataset.

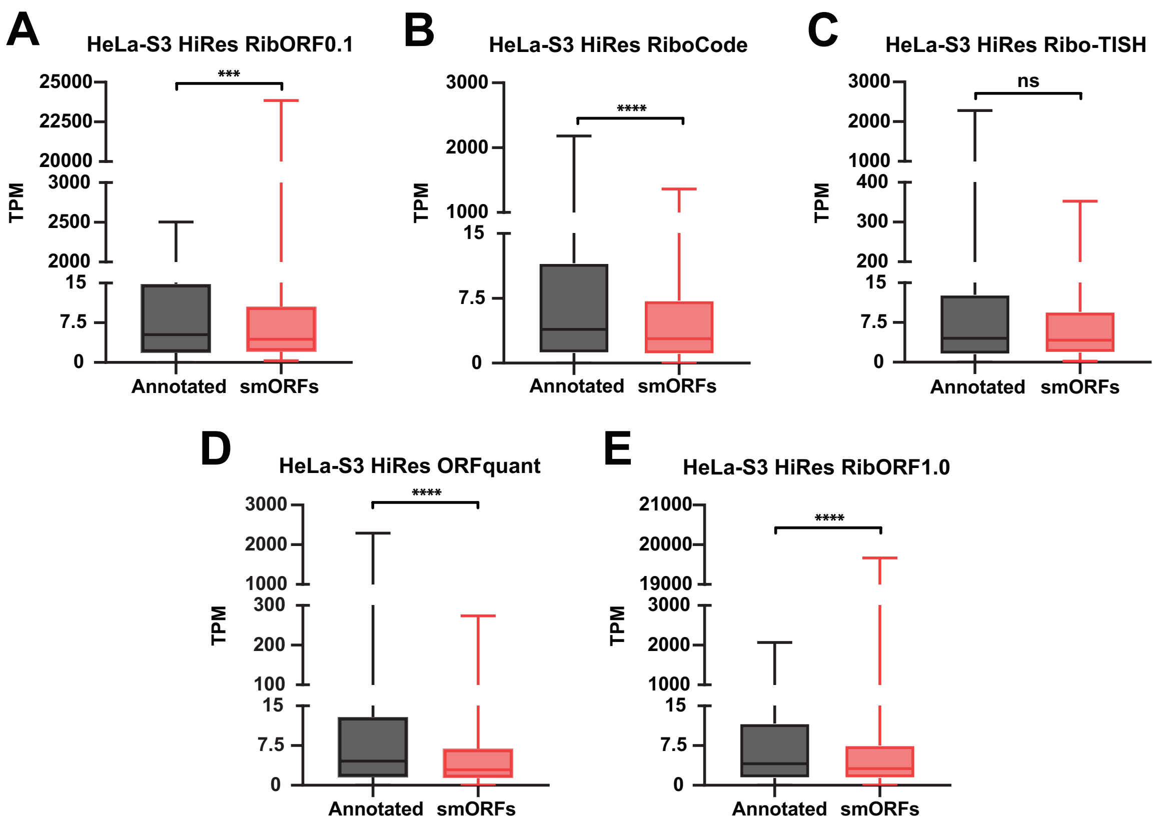

Supplementary Figure 11. Comparison of Ribo-seq coverage in annotated genes and smORFs called translated in HeLa-S3 high-resolution dataset. (A-E) Quantification of Ribo-seq read coverage for both annotated gene ORFs and smORFs called translated by each tool in the HeLa-S3 high-resolution dataset: RibORFv0.1 **(A)**, RiboCode **(B)**, Ribo-TISH **(C)**, ORFquant **(D)**, and RibORFv1.0 **(E)**. Coverage is calculated as transcripts per million (TPM) and are shown in Box-and-whisker plots. The box is bounded by the first and third quartiles, centerline shows the median, and whiskers represent the min and max values. Two-tailed Mann Whitney test was used to determine significant differences in coverage between annotated genes and smORFs (ns – not significant; ***, $p < 0.001$; ****, $p < 0.0001$).

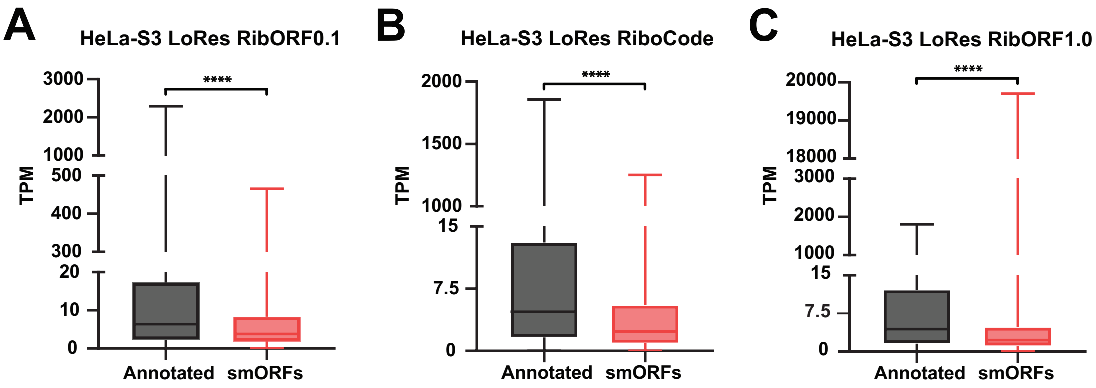

Supplementary Figure 12. Comparison of Ribo-seq coverage in annotated genes and smORFs called translated in HeLa-S3 low-resolution dataset. (A-C) Quantification of Ribo-seq read coverage for both annotated genes and smORFs called translated by each tool in the HeLa-S3 high-resolution dataset: RibORFv0.1 **(A)**, RiboCode **(B)**, and RibORFv1.0 **(C)**. Coverage is calculated as transcripts per million (TPM) and are shown in Box-and-whisker plots. The box is bounded by the first and third quartiles, centerline shows the median, and whiskers represent the min and max values. Two-tailed Mann Whitney test was used to determine significant differences in coverage between annotated genes and smORFs (****, $p < 0.0001$).

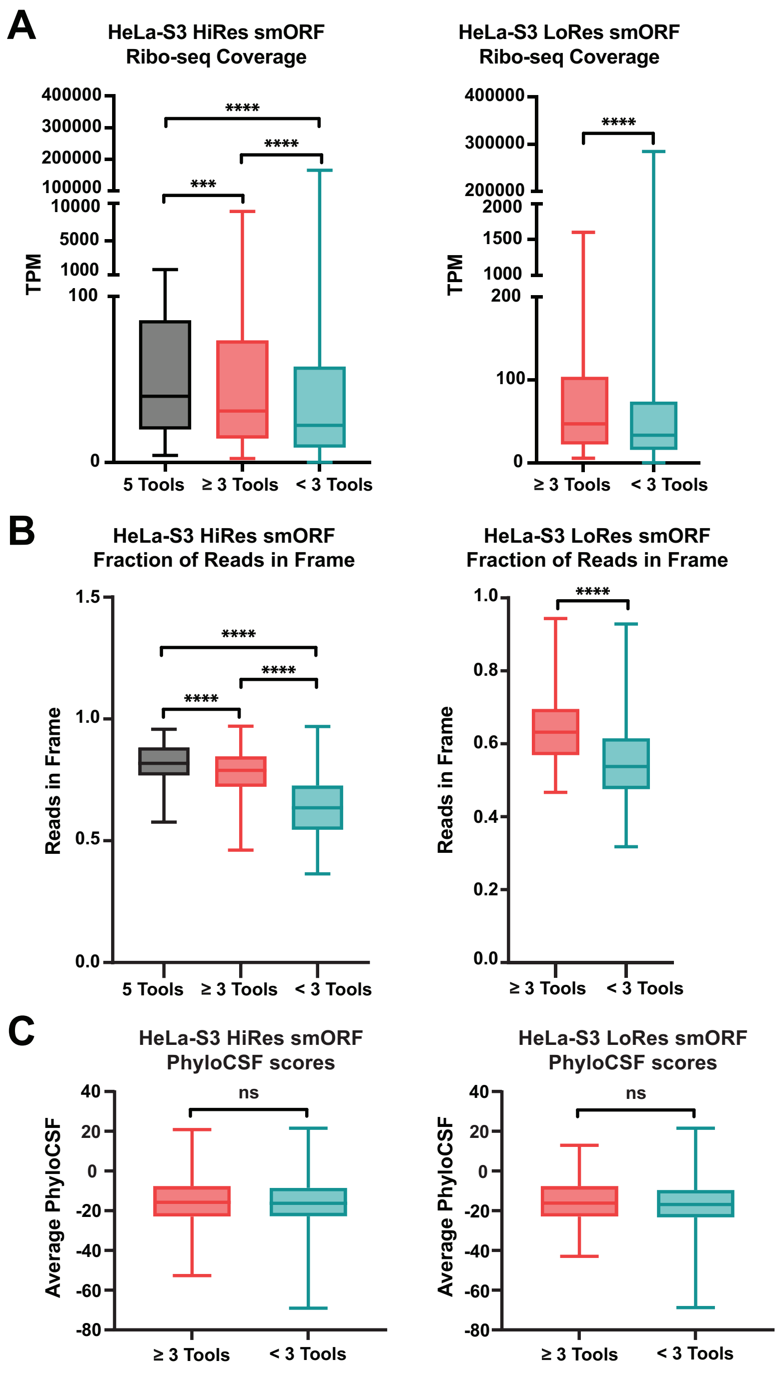

Supplementary Figure 13. smORFs called translated by multiple tools tend to have higher Ribo-seq coverage and higher fraction of reads in frame. (A) Box-and-whisker plots comparing Ribo-seq read coverage, calculated as transcripts per million (TPM), for smORFs called translated in high- and low-resolution HeLa-S3 datasets categorized as identified in all five tools, in greater than three tools, and less than three tools. The box is bounded by the first and third quartiles, centerline shows the median, and whiskers represent the min and max values. **(B)** Box-and-whisker plots comparing the fraction of Ribo-seq reads in frame for smORFs called translated in high- and low-resolution HeLa-S3 datasets categorized as identified in all five tools, in greater than three tools, and less than three tools. **(C)** Box-and-

whisker plots comparing average PhyloCSF scores of smORFs called translated in high- and low-resolution HeLa-S3 datasets categorized as identified in all five tools, in greater than three tools, and less than three tools. For all analyses, a two-tailed Mann Whitney test was used to determine significant differences (ns – not significant; ***, $p < 0.001$; ****, $p < 0.0001$).

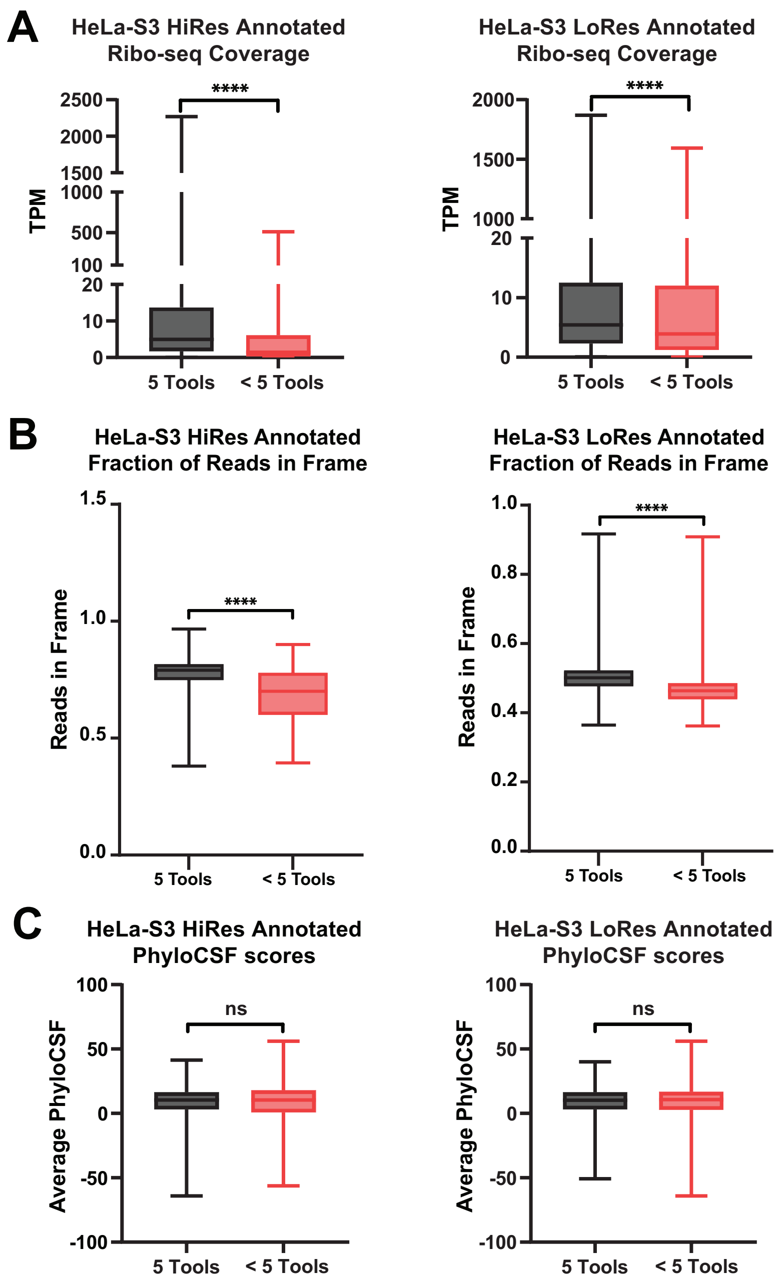

Supplementary Figure 14. Annotated genes called translated by all five tools tend to have higher Ribo-seq coverage and higher fraction of reads in frame. (A) Box-and-whisker plots comparing Ribo-seq read coverage, calculated as transcripts per million (TPM), for annotated genes called translated in high- and low-resolution HeLa-S3 datasets categorized as identified in all five tools or fewer than five tools. The box is bounded by the first and third quartiles, centerline shows the median, and whiskers represent the min and max values. **(B)** Box-and-whisker plots comparing the fraction of Ribo-seq reads in frame for annotated genes called translated in high- and low-resolution HeLa-S3 datasets categorized as identified in all five tools or fewer than five tools. **(C)** Box-and-whisker plots comparing average PhyloCSF scores of annotated genes called translated in high- and low-resolution HeLa-S3 datasets categorized as identified in all five tools or fewer than five tools. For all analyses, a two-tailed Mann Whitney test was used to determine significant differences (ns – not significant; ****, $p < 0.0001$).
